# Dysregulation of the BRCA1/long non-coding RNA NEAT1 signaling axis contributes to breast tumorigenesis

**DOI:** 10.18632/oncotarget.11364

**Published:** 2016-08-18

**Authors:** Pang-Kuo Lo, Yongshu Zhang, Benjamin Wolfson, Ramkishore Gernapudi, Yuan Yao, Nadire Duru, Qun Zhou

**Affiliations:** ^1^ Department of Biochemistry and Molecular Biology, Greenebaum Cancer Center, University of Maryland School of Medicine, Baltimore, MD 21201, USA

**Keywords:** BRCA1, NEAT1, miR-129, WNT4, breast cancer stem cells

## Abstract

Dysregulation of long non-codng RNA (lncRNA) expression has been found to contribute to tumorigenesis. However, the roles of lncRNAs in BRCA1-related breast cancer remain largely unknown. In this study, we delineate the role of the novel BRCA1/lncRNA NEAT1 signaling axis in breast tumorigenesis. BRCA1 inhibits NEAT1 expression potentially through binding to its genomic binding site upstream of the *NEAT1* gene. BRCA1 deficiency in human normal/cancerous breast cells and mouse mammary glands leads to NEAT1 overexpression. Our studies show that NEAT1 upregulation resulting from BRCA1 deficiency stimulates *in vitro* and *in vivo* breast tumorigenicity. We have further identified molecular mediators downstream of the BRCA1/NEAT1 axis. NEAT1 epigenetically silences miR-129-5p expression by promoting the DNA methylation of the CpG island in the *miR-129* gene. Silencing of miR-129-5p expression by NEAT1 results in upregulation of WNT4 expression, a target of miR-129-5p, which leads to activation of oncogenic WNT signaling. Our functional studies indicate that this NEAT1/miR-129-5p/WNT4 axis contributes to the tumorigenic effects of BRCA1 deficiency. Finally our *in silico* expression correlation analysis suggests the existence of the BRCA1/NEAT1/miR-129-5p axis in breast cancer. Our findings, taken together, suggest that the dysregulation of the BRCA1/NEAT1/miR-129-5p/WNT4 signaling axis is involved in promoting breast tumorigenesis.

## INTRODUCTION

Breast cancers have been classified into several molecular subtypes including luminal-A, luminal-B, HER2-positive, normal- and basal-like [1, 2]. Basal-like breast cancers (BLBCs) are frequently triple-negative for estrogen receptor (ER), progesterone receptor (PR) and human epidermal growth factor receptor 2 (HER2). BLBCs are an aggressive cancer with high tumor grade, increased proliferation rate, frequent recurrence, high metastatic rate, and the frequent presence of p53 mutations [2–4]. Patients with BLBCs have a poor prognosis and are difficult to treat [4]. Moreover, basal-like ductal carcinoma *in situ* (BL-DCIS) is known to be a potential precursor of invasive BLBCs [5, 6].

Breast cancer susceptibility gene 1 (BRCA1) encodes a multi-functional tumor suppressor protein that is necessary to maintain genomic integrity [7–11]. *BRCA1* germline mutations are one of the leading causes of hereditary breast and ovarian cancers [12, 13]. Strikingly, the majority of breast cancers that arise in BRCA1 mutation carriers manifest molecular phenotypes highly similar to basal-like/triple-negative breast cancers [3, 14–18]. BRCA1 is also functionally required for embryonic development and morphogenesis of mammary glands [19, 20]. However the molecular mechanisms underlying the BRCA1-dependent regulation of cell lineage differentiation and tumorigenesis remain elusive.

A large body of evidence demonstrates the existence of cancer stem cells (CSCs) in most types of cancer, including breast cancer. CSCs have stem-cell-like features and are shown to contribute to tumorigenesis, tumor heterogeneity, metastasis, and drug resistance in numerous types of cancer [21–24]. BLBCs are made up of a higher percentage of CSCs compared with breast cancers of other molecular subtypes [25, 26]. Due to the pivotal role of BRCA1 in mammary gland development and the large similarity between sporadic BLBCs and hereditary *BRCA1*-defective breast cancers, it has been postulated that BRCA1 deficiency attenuates breast CSC (BCSC) differentiation, resulting in accumulation of BCSCs in BLBCs [20]. However, the properties of BCSCs in BRCA1-defective breast cancers and the BRCA1-deficiency-triggered molecular alterations are still largely uncharacterized.

Long non-coding RNAs (lncRNAs) are key regulators of developmental processes, and their dysregulation is involved in cancer development and progression [27, 28]. The human *NEAT1* (Nuclear Enriched Abundant Transcript 1) gene encodes two lncRNA isoforms (3.7-kb NEAT1-1 and ∼23-kb NEAT1- 2) that play a central role in nuclear paraspeckles, which function in regulating RNA splicing and transcription [29]. *Neat1* has been reported to play a critical role in mouse mammary gland development [30]. NEAT1 functions as an oncogenic factor in multiple types of cancer, including breast cancer, and its expression is under the regulation of ERα signaling, the miR-449b-5p/c-Met axis, and hypoxia responses [31–34]. Recently, NEAT1 is reported to be involved in p53-triggered replication stress response and chemosensitivity [35]. These studies suggest that NEAT1 plays oncogenic roles in tumorigenic pathways and tumor responses to chemotherapy, warranting further investigations.

In this study, we have identified NEAT1 as a BRCA1-regulated lncRNA, and revealed the novel role of NEAT1 in BRCA1-deficiency-enhanced breast tumorigenesis.

## RESULTS

### BRCA1 inhibits the expression of the lncRNA NEAT1

Despite the critical roles of lncRNAs in developmental and tumorigenic regulation, their roles in BRCA1 function and its related diseases, in particular cancer, remain largely unknown. To date, only three lines of studies link BRCA1 to lncRNAs. BRCA1 has been reported to concentrate the lncRNA XIST on the inactive X chromosome to maintain its epigenetically silenced state via associating with XIST [36]. Another study reveals that BRCA1 can compete with the oncogenic lncRNA HOTAIR to bind EZH2, resulting in suppressing the functionality of EZH2-dependent polycomb-repressive complex 2 (PRC2) and PRC2-dependent gene expression regulation [37]. Finally, DDSR1 has been shown to be a BRCA1-binding lncRNA that is involved in DNA repair via stimulating homologous recombination [38].

**Figure 1:**
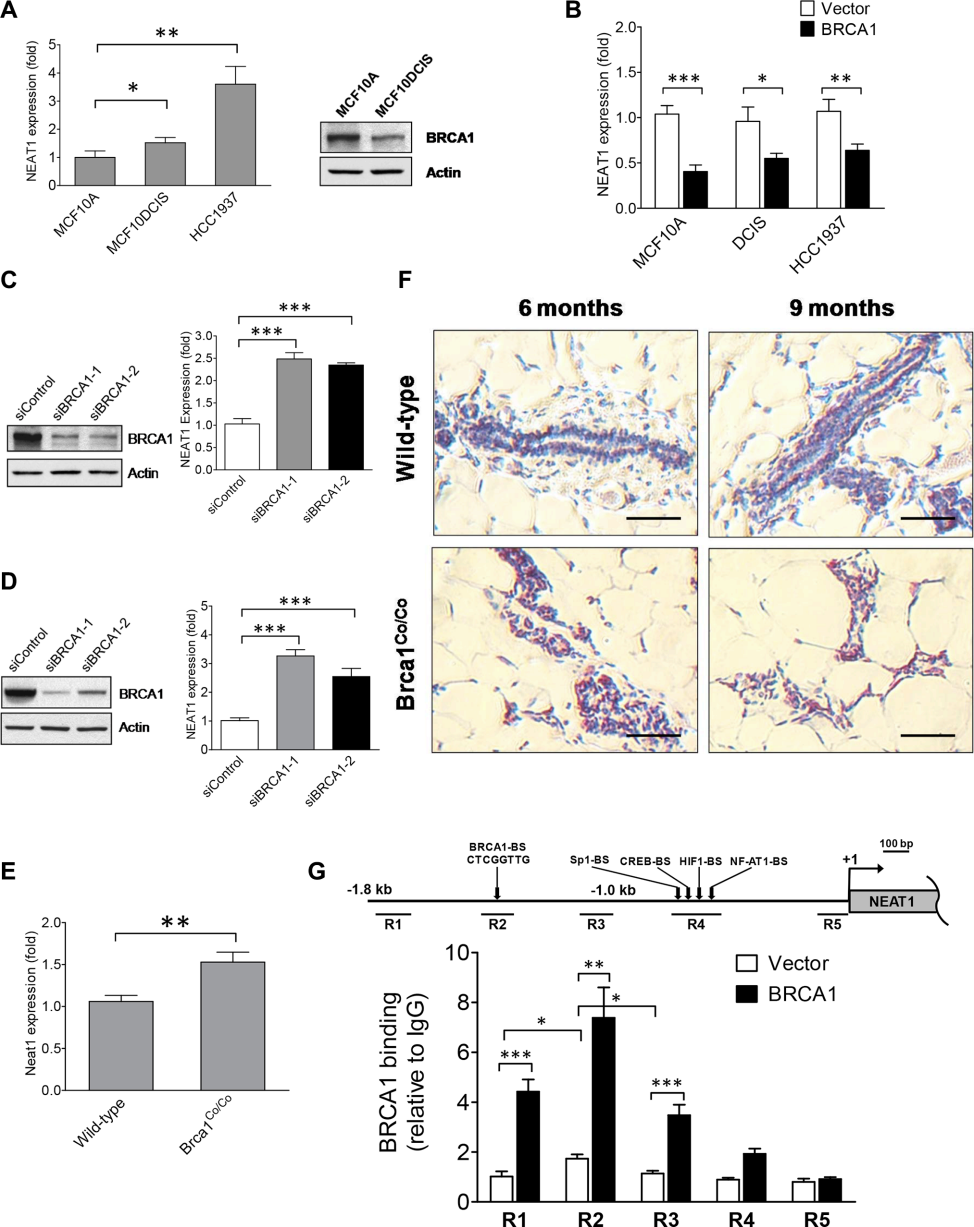
BRCA1 functions as an upstream regulator to inhibit the expression of the *NEAT1 gene* (**A**) Expression analysis of NEAT1 in MCF10A, MCF10DCIS and HCC1937 cells. qRT-PCR results are shown in the left panel. Western blot analysis of BRCA1 protein levels in MCF10A and MCF10DCIS cells is shown in the right panel. β-Actin protein levels were used as a protein loading control. (**B**) BRCA1 overexpression downregulates NEAT1 expression. qRT-PCR analysis of NEAT1 expression was performed on MCF10A, MCF10DCIS and HCC1937 cells transfected with the empty vector or BRCA1 expression plasmid DNA. (**C**, **D**) BRCA1 knockdown leads to induction of NEAT1 expression. Western blot analysis of BRCA1 and β-Actin (left panels) and qRT-PCR analysis of NEAT1 expression (right panels) were performed on MCF10A (C) and MCF7 (D) cells transfected with either the control or BRCA1 siRNA. Two different BRCA1 siRNAs (siBRCA1-1 and siBRCA1-2) were used in the knockdown experiment. (**E**) Expression analysis of Neat1 RNA levels in wild-type and Brca1-deficient mammary glands. qRT-PCR analysis of Neat1 expression was performed on mammary glands from wild-type and Brca1-deficient (*Brca1*^Co/Co^) mice. (**F**) Brca1 deficiency gives rise to elevated Neat1 RNA levels in ductal epithelial cells of mammary glands. *In situ* hybridization (ISH) analysis of Neat1 RNA expression was performed on mammary gland tissue sections from wild-type and *Brca1*^Co/Co^ mice. The scale bar indicates 50 μm. (**G**) BRCA1 protein binds its cognate binding site *in vivo* located in the upstream genomic region of the human *NEAT1* gene. ChIP assays using the BRCA1 antibody or the mouse IgG control were performed on MCF10A cells transfected with the control empty vector or BRCA1 expression plasmid DNA. qPCR assays were performed on ChIP samples to quantitate five distinct immunoprecipitated genomic DNA regions (R1 to R5 as indicated in the map) upstream of the *NEAT1* gene by using five different pairs of primers. The genomic map for the 5′-end of the *NEAT1* gene and its upstream DNA region is shown in the top panel. The putative transcription factor binding sites are depicted in the map. The result of quantitative ChIP analysis is presented as a bar graph shown in the bottom panel. Ectopically expressed BRCA1 protein predominantly bound to the R2 region. An approximately two-fold increase in the binding to the R2 region in the empty-vector-transfected cell sample was derived from the binding of endogenous BRCA1 protein. For all of bar graphs presented here, the error bar represents the standard deviation (SD) of the dataset (*n* = 3). *p < 0.05, **p < 0.01, ***p < 0.001.

Due to the critical roles of both BRCA1 and the lncRNA NEAT1 in epigenetic regulation and oncogenesis, we hypothesized that NEAT1 may play a role in the BRCA1-dependent signaling pathway. To test this hypothesis, we examined the correlation between BRCA1 status and NEAT1 expression in the immortalized human mammary epithelial cell (HMEC) line MCF10A, BL- DCIS cell line MCF10DCIS [39–41] and BLBC cell line HCC1937. While both MCF10A and MCF10DCIS express wild-type BRCA1, HCC1937 is a model of BRCA1-deficiency breast cancer wherein one allele is mutated while the other is deleted. NEAT1 expression levels were moderately elevated in MCF10DCIS and highly upregulated in HCC1937 cells when compared with the HMEC control MCF10A (Figure 1A). Given that HCC1937 cells are BRCA1-deficient, this result suggested a possible relationship between BRCA1 inactivation and upregulation of NEAT1 expression. To determine if NEAT1 upregulation in MCF10DCIS cells correlates with decreased BRCA1 expression levels, we examined the protein levels of BRCA1 in MCF10DCIS and MCF10A cells. Western blot result showed that BRCA1 protein levels were moderately decreased in MCF10DCIS cells compared to MCF10A cells (Figure 1A), correlating with elevated NEAT1 expression levels.

To reveal the role of BRCA1 in NEAT1 expression regulation, we overexpressed BRCA1 in MCF10A, MCF10DCIS, and HCC1937 cells and examined NEAT1 expression. Western blot analysis confirmed that BRCA1 plasmid-transfected cells expressed high levels of the ectopic BRCA1 protein (Supplementary Figure S1). As shown in Figure 1B, ectopic expression of BRCA1 downregulated total NEAT1 RNA levels in these three cell lines compared to control empty-vector-transfected cells, indicating that BRCA1 overexpression inhibits NEAT1 RNA expression. To verify this finding, we performed BRCA1 knockdown experiments using two different siRNAs [42, 43] on MCF10A and the luminal breast cancer cell line MCF7, which both express wild-type BRCA1. Both BRCA1 siRNAs (siBRCA1-1 and siBRCA1-2) effectively depleted BRCA1 protein expression (Figure 1C and 1D). BRCA1 knockdown resulted in significant upregulation of total NEAT1 expression levels in both cell lines (Figure 1C and 1D). We also examined the expression level of each specific NEAT1 isoform (NEAT1- 1 and NEAT1-2) in BRCA1-knockdown cells and confirmed that BRCA1 knockdown led to upregulation of both NEAT1 isoforms (Supplementary Figure S2). Gain- and loss-of-function studies, taken together, indicate that BRCA1 negatively regulates NEAT1 expression.

To further validate the inhibitory effect of BRCA1 on NEAT1 expression *in vivo*, we used qRT-PCR to analyze Neat1 RNA expression in wild-type and Brca1-deficient mouse mammary glands. Brca1-deficient mammary glands were isolated from mammary tumor virus *(MMTV)-Cre;Brca1* conditional exon 11 deletion (*Brca1*^Co/Co^) mice that express exon 11-deleted Brca1 instead of full-length Brca1 in *MMTV*-active tissues (including mammary glands) and exhibit a tumorigenic phenotype in mammary gland tissues [44, 45]. As shown in Figure 1E, Neat1 RNA expression was higher in Brca1-deficient mammary glands from *MMTV-Cre;Brca1*^Co/Co^ mice than in mammary glands from control wild-type mice (*C57BL/6*). To confirm the qRT-PCR result and examine which cell types express Neat1 RNA, we performed *in situ* hybridization assays (ISH) on paraffin-embedded mammary gland tissue sections from wild-type and *MMTV-Cre;Brca1*^Co/Co^ mice with 6 and 9 months of age. The ISH result showed that Neat1 was predominantly expressed in nuclei of ductal cells, and Neat1 staining was stronger in *Brca1*^Co/Co^ mammary tissue than in wild-type counterpart tissue (Figure 1F). These results suggest that NEAT1 upregulation by BRCA1 deficiency may be physiologically relevant in mice and humans.

In addition to its critical role in DNA damage repair, BRCA1 is a transcriptional regulator that can activate and repress gene expression [46]. While BRCA1 can physically associate with other transcription factors (e.g. c-Myc) to regulate gene expression [46], Cable *et al.* found that BRCA1 protein complexes are also able to regulate gene expression by directly binding to DNA sequences containing the pattern “TTC(G/T)GTTG” [47]. We searched the upstream genomic sequence of the human *NEAT1* gene and identified a putative BRCA1-binding site with the sequence “CTCGGTTG” 1.4-kb upstream of the *NEAT1* gene (Figure 1G). This suggested that *NEAT1* may be a direct target gene of BRCA1. To test this possibility, we performed chromatin immunoprecipitation (ChIP) assays to examine the direct binding of BRCA1 to the upstream sequence region of *NEAT1*. The ChIP qPCR data showed that the binding of ectopic and endogenous BRCA1 protein was significantly enriched at the genomic DNA region containing the putative BRCA1-binding site (*n* = 3; *p* < 0.05 for endogenous enrichment; *p* < 0.01 for ectopic enrichment) (Figure 1G). These data suggest that BRCA1 negatively regulates NEAT1 expression potentially through binding to its cognate DNA site upstream of the *NEAT1* gene.

### NEAT1 is functionally required for malignancies and stemness of breast tumor cells

**Figure 2:**
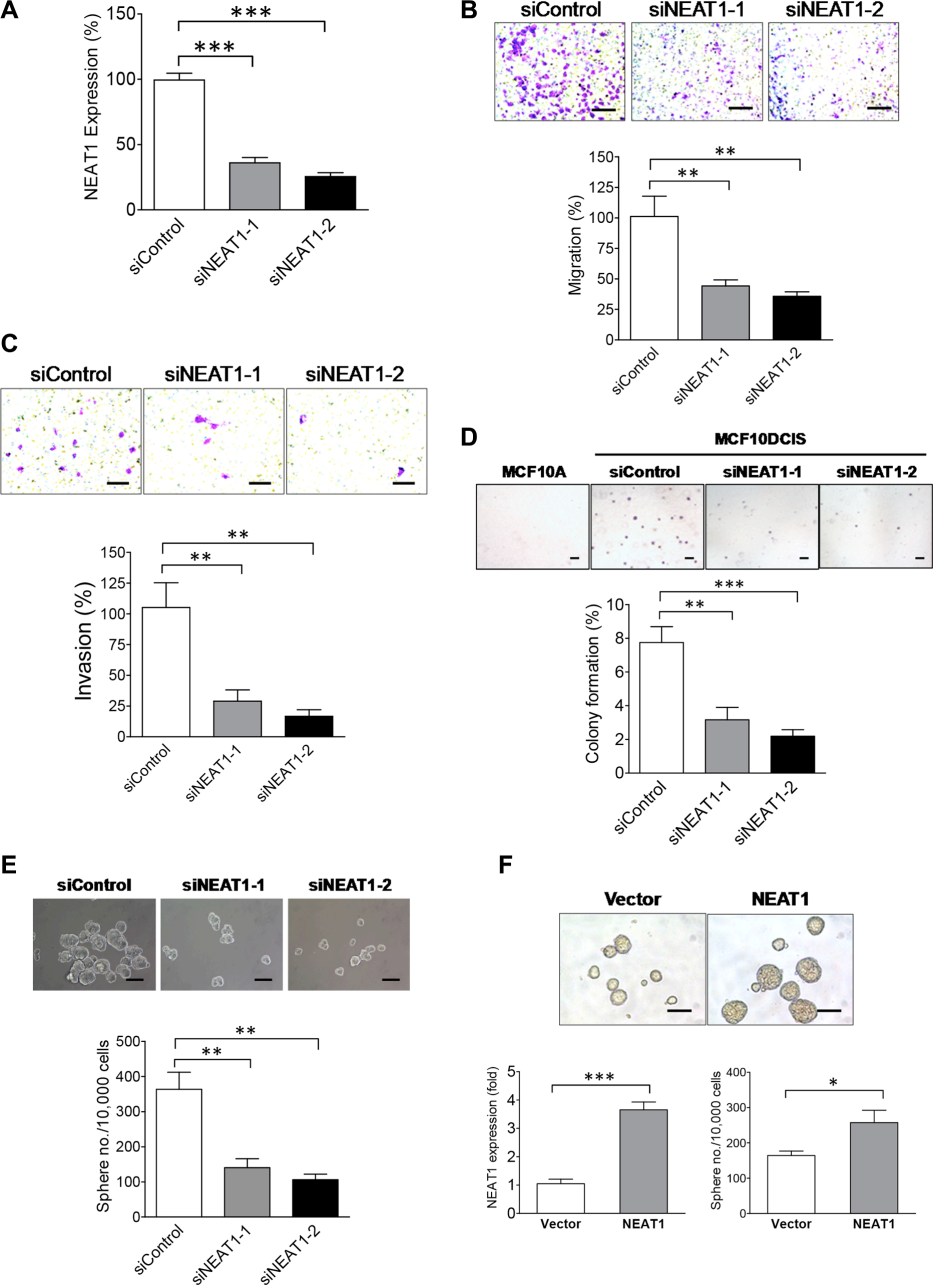
NEAT1 is an oncogenic factor required for invasiveness, anchorage-independent growth and stemness of MCF10DCIS tumor cells **(A)** The knockdown efficiency of two NEAT1 siRNAs. qRT-PCR analysis of NEAT1 expression was performed on MCF10DCIS cells transfected with the control or two distinct NEAT1 siRNAs. (**B)** NEAT1 knockdown suppresses the migratory ability of MCF10DCIS tumor cells. Transwell migration assays were performed on MCF10DCIS cells transfected with the control or two different NEAT1 siRNAs for 48 hours. The stained pictures are shown in the top panel and the quantitative bar graph data (*n* = 3) are shown in the bottom panel. The scale bar indicates 100 μm. (**C)** NEAT1 knockdown inhibits the invasive ability of MCF10DCIS tumor cells. Invasion assays were performed on MCF10DCIS cells transfected with the control or two different NEAT1 siRNAs for 48 hours. The stained pictures are shown in the top panel and the quantitative bar graph data (*n* = 3) are shown in the bottom panel. The scale bar indicates 100 μm. (**D)** NEAT1 knockdown inhibits the anchorage-independent growth of MCF10DCIS tumor cells. Soft agar assays were performed on MCF10DCIS cells transfected with the control or two different NEAT1 siRNAs for 48 hours. MCF10A cells were also included in assays to serve as a non-malignant cell control. The stained pictures are shown in the top panel and the quantitative bar graph data (*n* = 3) are shown in the bottom panel. The scale bar indicates 200 μm. (**E)** NEAT1 knockdown results in the decreased self-renewal and proliferation of BCSCs in MCF10DCIS cells. Stem-cell sphere formation assays were performed on MCF10DCIS cells transfected with the control or two NEAT1 siRNAs for 48 hours. Pictures of BCSC spheres are shown in the top panel and sphere formation efficiency data (*n* = 3) are shown in the bottom panel. The scale bar indicates 100 μm. (**F)** NEAT1 overexpression enhances the self-renewal of breast stem cells. Stem-cell sphere formation assays (the bottom-right panel) and qRT-PCR analysis of NEAT1 expression (the bottom-left panel) were performed on MCF10A cells transfected with the empty vector or NEAT1 expression plasmid DNA for 24 hours. Pictures of breast stem-cell spheres are shown in the top panel and the scale bar indicates 100 μm. The error bar in bar graphs represents the SD of the dataset (*n* = 3). *p < 0.05, **p < 0.01, ***p < 0.001.

NEAT1 has been shown to play an oncogenic role in prostate tumorigenesis and hypoxia-related breast cancer cell survival [31, 32]. To reveal the functional role of NEAT1 in breast cancer, we conducted a series of functional studies to examine the role of NEAT1 in the invasiveness, anchorage-independent growth and stemness of breast cancer cells. As shown in Figure 2A, two different NEAT1 siRNAs [31] effectively knockdowned NEAT1 expression in MCF10DCIS cells. By performing transwell migration and invasion studies, we found that NEAT1 knockdown substantially suppressed the migratory and invasive ability of MCF10DCIS cells (Figure 2B and 2C). Moreover, soft agar assays showed that NEAT1 is required for the anchorage-independent growth of MCF10DCIS cells as NEAT1 knockdown significantly suppressed the colony formation of MCF10DCIS cells in soft agar (Figure 2D).

We next evaluated the effect of NEAT1 knockdown on BCSCs in MCF10DCIS cells by stem-cell sphere formation assays. Depletion of NEAT1 by both siRNAs attenuated self-renewal and the proliferation rate of BCSCs in MCF10DCIS cells, indicated by the decreased number and reduced size of BCSC spheres (Figure 2E). In contrast, ectopic expression of the short isoform of 3.7-kb NEAT1 enhanced the mammosphere formation of MCF10A cells, indicating that NEAT1 overexpression gave rise to increased stemness of MCF10A cells (Figure 2F). These gain- and loss-of-function studies show that NEAT1 is functionally required for invasiveness, anchorage-independent growth and BCSC self-renewal of breast tumor cells and its overexpression leads to increased self-renewal of normal breast stem cells. The enhancing effect of NEAT1 on BCSCs is not restricted to MCF10DCIS cells as NEAT1 is also crucial for maintaining self-renewal of BCSCs in basal and luminal breast cancer cell lines HCC1937 and MCF7, evidenced by the inhibitory effect of NEAT1 knockdown on the BCSC sphere formation of HCC1937 and MCF7 cells (Supplementary Figures S3 and S4). These findings suggest that NEAT1 plays an oncogenic role in breast cancer stem cells.

### NEAT1 is crucial for enhanced tumorigenic phenotypes and stemness of breast tumor cells with BRCA1 deficiency

**Figure 3:**
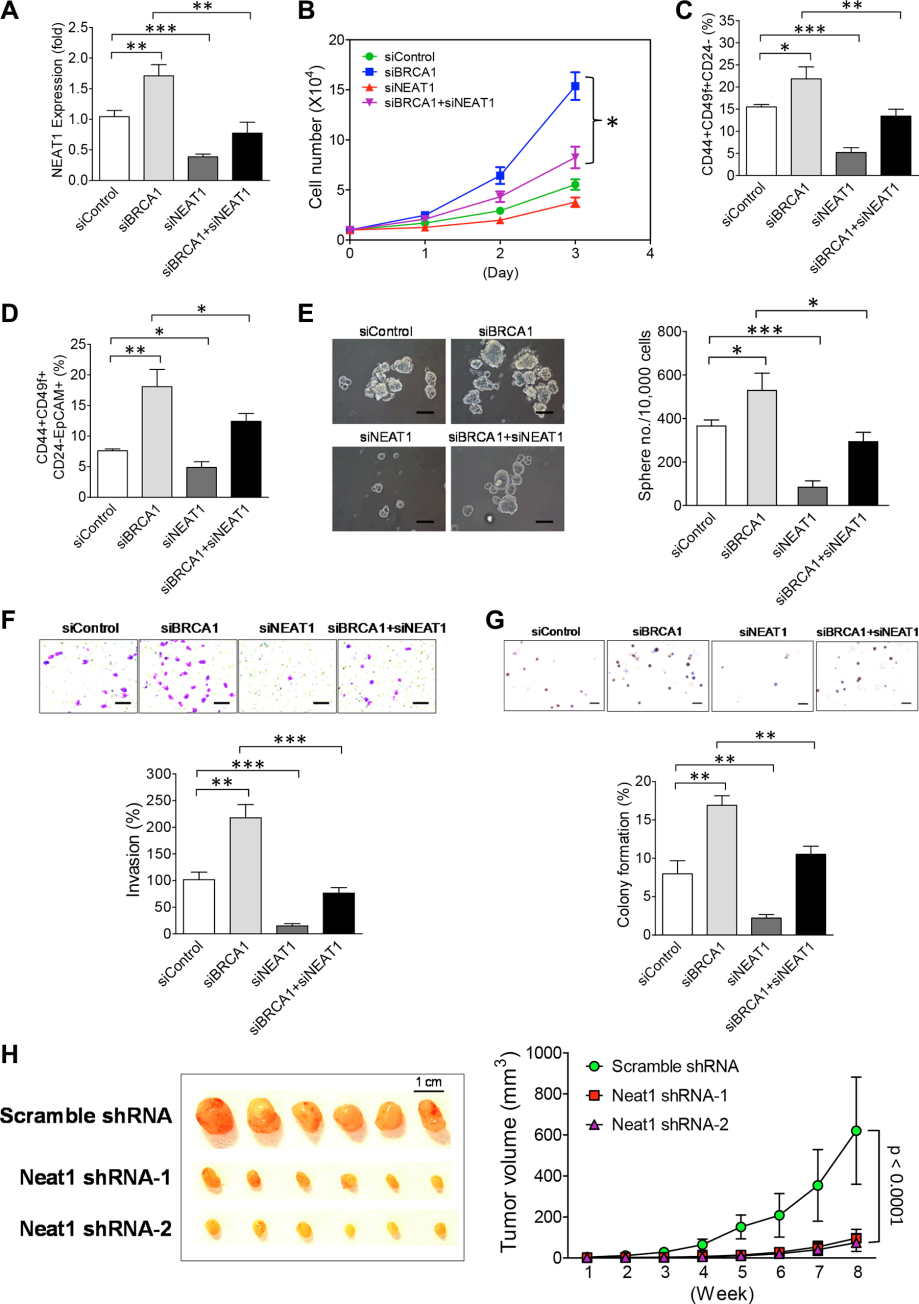
NEAT1 upregulation induced by BRCA1 deficiency promotes *in vitro* malignancies and *in vivo* tumorigenicity of breast tumor cells (**A**) NEAT1 siRNA abolishes the upregulation of NEAT1 by BRCA1 knockdown. qRT-PCR analysis of NEAT1 expression was performed on MCF10DCIS cells transfected with the control siRNA (siControl), the siRNA targeting BRCA1 (siBRCA1-1) or NEAT1 (siNEAT1-2), or the siRNA combination targeting both genes for 48 hours. (**B**) Co-knockdown of NEAT1 partially abolishes the enhanced proliferation of BRCA1-knockdown MCF10DCIS cells. At 24 hrs after transfection, 10,000 siRNA-transfected MCF10DCIS cells shown in (A) were seeded for cell proliferation assays. Live cell counting was performed by trypan blue dye exclusion assays. (**C**) Co-knockdown of NEAT1 attenuates the expansion of BCSCs in BRCA1-knockdown MCF10DCIS cells. Flow cytometry analysis of surface antigen markers CD44, CD49f and CD24 for BCSC identification was performed on siRNA-transfected MCF10DCIS cells shown in (A). The 2D dot plots that profile CD44+CD49f+ and CD44+CD49f+CD24– cell subsets are shown in Supplementary Figure S7A. The percentages of BCSC-enriched CD44+CD49f+CD24– cell subsets from three independent flow cytometry experiments were used to make the bar graph. Error bars indicate standard deviation (SD). (**D**) BRCA1 knockdown significantly expands the EpCAM+BCSC population and co-knockdown of NEAT1 attenuates its expansion. Flow cytometry analysis of BCSC-related protein antigens CD44, CD49f, CD24 and EpCAM was performed on siRNA-transfected MCF10DCIS cells shown in (A). The gated CD44+CD49f+ cell subsets shown in Supplementary Figure S7A were subjected to the 2D dot plot analyses that profile CD24 and EpCAM as shown in Supplementary Figure S7B. The percentages of EpCAM+BCSC cell subsets (CD44+CD49f+CD24–EpCAM+) in siRNA-transfected MCF10DCIS cells from three independent flow cytometry experiments were used to make the bar graph. (**E**) Co-knockdown of NEAT1 impairs BRCA1-knockdown-induced increases in the size and formation efficiency of BCSC spheres developed from MCF10DCIS cells. At 48 hrs after transfection, siRNA-transfected MCF10DCIS cells shown in (A) were seeded in six-well plates for stem-cell sphere formation assays. Pictures of formed BCSC spheres are shown in the left panel and the bar graph of sphere formation efficiency data is shown in the right panel. The scale bar indicates 100 μm. (**F**) NEAT1 knockdown abolishes a BRCA1-deficiency-induced increase in invasiveness of MCF10DCIS tumor cells. 48 hours posttransfection, siRNA-transfected MCF10DCIS cells shown in (A) were subjected to invasion assays. The stained pictures are shown in the top panel and the quantitative bar graph of invasion data is shown in the bottom panel. The scale bar indicates 100 μm. (**G**) Co-knockdown of NEAT1 attenuates the increased anchorage-independent growth of MCF10DCIS tumor cells induced by BRCA1 knockdown. 48 hours posttransfection, soft agar assays were performed on siRNA-transfected MCF10DCIS cells shown in (A). The stained pictures are shown in the top panel and the quantitative bar graph of colony formation efficiency data is shown in the bottom panel. The scale bar indicates 200 μm. (**H**) Neat1 knockdown inhibits the *in vivo* development of Brca1-deficient xenograft mammary tumors. 1 × 10^6^ stable scramble shRNA-expressing or shNeat1-expressing tumor cells that were derived from Brca1-deficient mammary tumors developed in *MMTV-cre;Brca1*^co/co^ mice were injected into the fourth mammary fat pad of syngeneic female mice. The development of xenograft tumors was monitored for eight weeks and tumor size was measured weekly. Tumor growth curves (*n* = 6) were plotted and are shown in the right panel. The dissected tumors were photographed and are shown in the left panel. The knockdown efficiency of these two Neat1 shRNAs (shNeat1-1 and shNeat1-2) is shown in Supplementary Figure S6A. The error bar shown in all bar graphs and the growth rate plot represents the SD of the dataset (*n* = 3). *p < 0.05, **p < 0.01, ***p < 0.001.

Inactivation of BRCA1 has been shown to promote proliferation of mammary epithelial cells and the expansion of mammary stem/progenitor cells via suppression of their differentiation [48–53]. To decipher the role of NEAT1 in BRCA1-deficiency-driven breast tumorigenesis, we performed NEAT1 and BRCA1 co-knockdown studies in MCF10DCIS cells. Given that BRCA1 knockdown led to upregulation of NEAT1 (Figure 1C and 1D), we first examined whether co-knockdown of NEAT1 can abolish this upregulation. Using qRT-PCR we demonstrated that NEAT1 siRNA-mediated knockdown was sufficient to abolish the BRCA1-knockdown-mediated upregulation of NEAT1 expression (Figure 3A). The qRT-PCR result was further confirmed by Western blot analysis showing that co-transfection of NEAT1 siRNA (siNEAT1-2) had no significant, interfering effect on the knockdown efficiency of BRCA1 siRNA (siBRCA1-1) (Supplementary Figure S5). While knockdown of BRCA1 increased cell growth as previously reported [48–53], co-knockdown of NEAT1 suppressed approximately 70% of the increased cell growth induced by BRCA1 knockdown (Figure 3B). These results suggest that the BRCA1-deficiency-enhanced proliferation of MCF10DCIS cells relies, at least in part, on NEAT1.

Our previous studies showed that BCSCs of the BL- DCIS cell line MCF10DCIS possess the stem-cell surface marker profile CD44+CD49f+CD24– [54–56]. To reveal the roles of BRCA1 and NEAT1 in BCSCs, we performed flow cytometry analysis of these three stem-cell markers in MCF10DCIS cells with either single or double knockdown of BRCA1 and NEAT1. BRCA1 knockdown increased the BCSC population (21.85 ± 2.71% vs. 15.51 ± 0.55% of the control siRNA, *p* < 0.05; *n* = 3), whereas NEAT1 knockdown dramatically reduced the BCSC population (5.21 ± 1.08% vs. 15.51 ± 0.55% of the control siRNA, *p* < 0.001; *n* = 3) (Figure 3C). Remarkably, co-knockdown of NEAT1 abolished the BRCA1-knockdown-mediated enhancement of the BCSC population in MCF10DCIS cells (from 21.85 ± 2.71% down to 13.43 ± 1.56% relative to 15.51 ± 0.55% of the control siRNA, *p* < 0.01; *n* = 3) (Figure 3C). These findings demonstrate that NEAT1 upregulation is required for the expansion of BCSCs in MCF10DCIS cells when BRCA1 is inactivated.

It has been reported that haplodeficiency of BRCA1 induces the expansion of the luminal mammary progenitor population (CD49f+EpCAM+) [50, 52], suggesting that luminal mammary progenitor cells possess a molecular signature of CD49f+EpCAM+ and BRCA1 is involved in promoting their differentiation. Recently Pandey *et al.* have also identified BCSCs in the MCF10DCIS cell line using stem cell surface protein markers (CD44+ESA+CD24–; ESA is the alternative name of EpCAM) [57]. These two lines of studies suggest that EpCAM is a potential stem cell marker for MCF10DCIS cells, especially when BRCA1 is deficient. To reveal whether BRCA1 inactivation in MCF10DCIS cells can affect EpCAM+ and EpCAM– BCSC populations, we repeated our analysis of the FACS data shown in Figure 3C with the addition of the luminal progenitor marker EpCAM. We sorted the CD44+CD49f+CD24– cell population into two distinct cell subsets according to the EpCAM expression status, CD44+CD49f+CD24–EpCAM+ and CD44+CD49f+CD24–EpCAM–. Consistent with reported findings from *BRCA1*-mutated patients, BRCA1 knockdown increased the CD44+CD49f+CD24–EpCAM+ (EpCAM+BCSC) cell subset (18.07 ± 2.83% vs. 7.62 ± 0.28% of the control siRNA, *p* < 0.05; *n* = 3) while NEAT1 knockdown led to a significant decrease in the EpCAM+BCSC subset (4.87 ± 0.95% vs. 7.62 ± 0.28% of the control, *p* < 0.01; *n* = 3) (Figure 3D). The effect of NEAT1 knockdown on BCSCs was confirmed by a second NEAT1 siRNA (siNEAT1-1) [31] (Supplementary Figure S8). Co-knockdown of NEAT1 partially abolished the BRCA1 knockdown-induced expansion of the EpCAM+BCSC population (from 18.07 ± 2.83% down to 12.42 ± 1.28%, *p* < 0.05; *n* = 3), but was not sufficient to decrease the percentage of this EpCAM+BCSC population to the control level (12.42 ± 1.28% vs 7.62 ± 0.28% in the control siRNA) (Figure 3D). To validate these flow cytometry data, we performed stem-cell sphere formation assays on single-knockdown and co-knockdown cells. In line with flow cytometry results, NEAT1 knockdown suppressed the effect of BRCA1 knockdown on enhancing self-renewal and the proliferation rate of BCSCs, indicated by the decreased sphere number and reduced sphere size (Figure 3E). Consistent with flow cytometry and sphere data, co-knockdown of NEAT1 suppressed BRCA1-knockdown-induced increases in invasion and anchorage-independent growth of MCF10DCIS cells (Figure 3F and 3G). These results suggest that abrogation of BRCA1 function suppresses the differentiation of EpCAM+BCSCs and promotes their expansion and malignancies. Moreover, we found that NEAT1 upregulation is required for the BRCA1-deficiency-driven effect on increasing the EpCAM+BCSC population and enhancing malignancies of breast cancer cells.

To determine if NEAT1 is functionally required for the *in vivo* tumorigenicity of BRCA1-deficienct breast tumor cells, we isolated primary tumor cells from Brca1-deficient mammary tumors developed in *MMTV-Cre;Brca1*^Co/Co^ mice and established stable Neat1 knockdown in primary *Brca1* mutant mammary tumor cell cultures as described in Supplementary Figure S6. Two different Neat1 shRNAs we used effectively knockdowned endogenous mouse Neat1 RNA levels (Supplementary Figure S6A). Consistent with findings from human breast tumor cells (Figure 3E), Neat1 knockdown suppressed *in vitro* self-renewal of CSCs in mouse *Brca1* mutant mammary tumors (Supplementary Figure S6B). To test the effect of Neat1 knockdown on Brca1-deficient mammary tumor development, we perform *in vivo* tumorigenicity analysis on scramble (as a control) and Neat1 shRNA transfectants of primary tumor cell cultures. As shown in Figure 3H, Neat1 knockdown by two different Neat1 shRNAs significantly impaired the development of Brca1-deficient xenograft mammary tumors. This *in vivo* finding strongly supports that NEAT1 is functionally required for breast tumorigenesis activated by BRCA1 deficiency.

### Epigenetic silencing of tumor-suppressor miR- 129-5p by NEAT1

**Figure 4:**
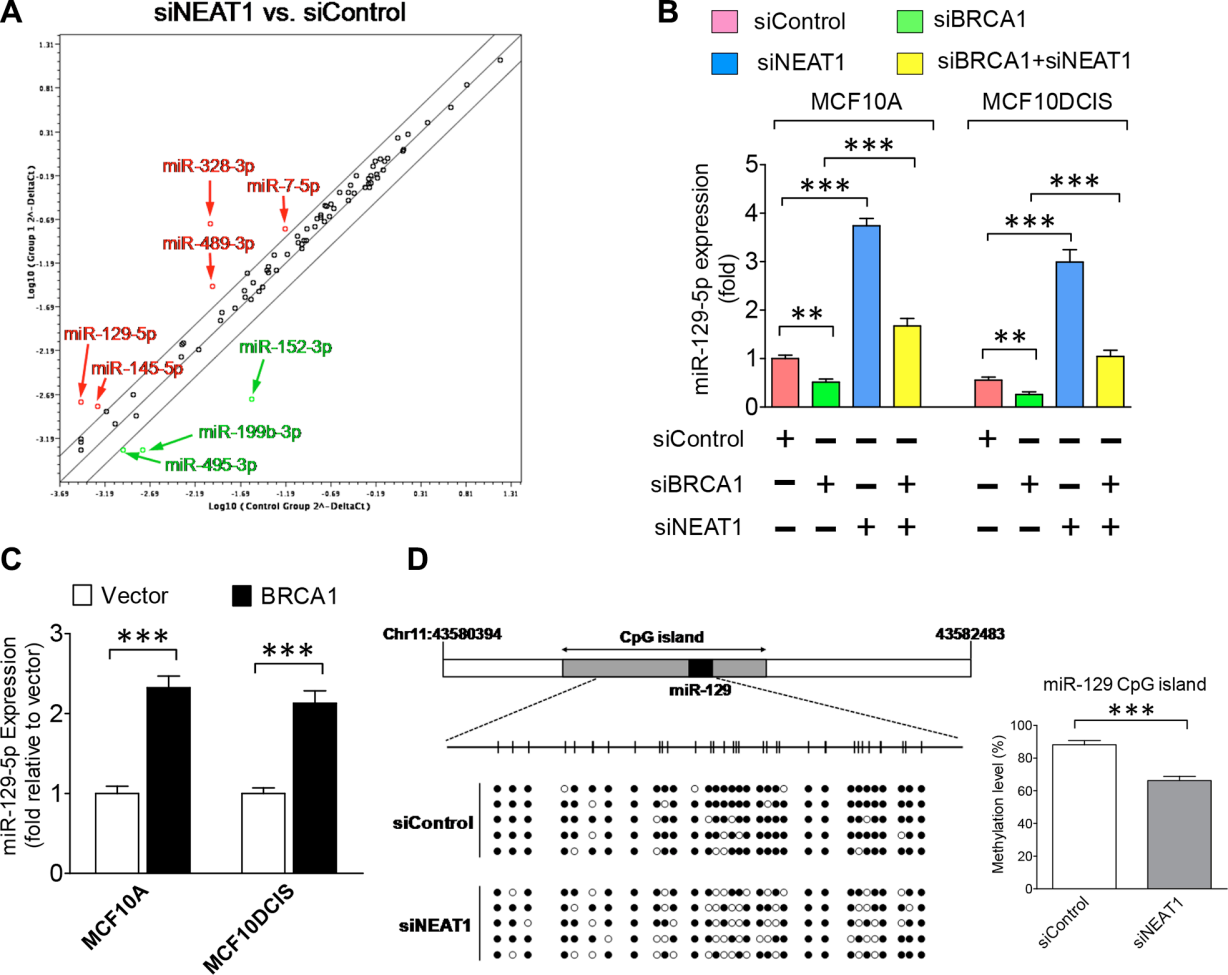
Epigenetic regulation of miR-129-5p expression by the BRCA1/NEAT1 axis (**A**) Expression profiling of miRNAs in MCF10A cells with NEAT1 knockdown by PCR array assays. The common logarithms of miRNA expression values from control siRNA-transfected cells were plotted against those from NEAT1 siRNA (siNEAT1-2)-transfected cells to make the scatter plot. miRNAs that were upregulated and downregulated at least 2-fold in NEAT1-knockdown MCF10A cells are indicated by the red and green colors, respectively. These identified NEAT1-regulated miRNAs were confirmed by another NEAT1 siRNA (siNEAT1-1) (data not shown). (**B**) Downregulation of miR-129-5p expression by BRCA1 knockdown is abolished by co-knockdown of NEAT1. qRT-PCR analysis of miR-129-5p expression was performed on MCF10A and MCF10DCIS cells transfected with the control siRNA, the siRNA targeting BRCA1 or NEAT1, or the siRNA combination targeting both genes for 48 hours. (**C**) Ectopic expression of BRCA1 upregulates miR- 129- 5p expression. qRT-PCR analysis of miR-129-5p expression was performed on MCF10A and MCF10DCIS cells transfected with the empty vector or BRCA1 expression plasmid DNA. (**D**) NEAT1 knockdown leads to partial demethylation of the CpG island in the *miR-129* gene. Bisulfite sequencing analysis of the DNA region (containing 32 CpG dinucleotides) within the CpG island of the *miR-129* gene was performed on genomic DNA samples isolated from control and NEAT1 siRNA-transfected MCF10DCIS cells. Filled and unfilled circles represent methylated and unmethylated CpG dinucleotides, respectively. The methylation level bar graph with error bars was plotted based on sequencing results from five randomly selected clones for each sample. The error bar shown in sub-figures (B, C) represents the SD of the dataset (*n* = 3). **p < 0.01, ***p < 0.001.

NEAT1 has been shown to regulate gene expression by serving as a splicing regulator, modulator of chromatin remodeling and microRNA sponge [29, 31, 34, 58]. MicroRNAs (miRNAs) have been shown to play critical roles in CSC regulation [59, 60], and to interact with lncRNAs in non-coding RNA-regulatory networks [61]. To decipher the molecular mechanism underlying the oncogenic effect of NEAT1, we performed miRNA profiling analysis. Using miRNA PCR arrays that detect 84 breast cancer-related miRNAs, we identified eight differentially expressed miRNAs in NEAT1-knockdown MCF10A cells, including five upregulated miRNAs (miRs-7-5p, -129-5p, -145-5p, -328-3p, -489-3p) and three downregulated miRNAs (miRs-152-3p, -199b-3p, -495-3p) (Figure 4A).

Among these NEAT1-regulated miRNAs, miR- 129- 5p was of particular interest as it was previously reported to be epigenetically silenced in breast and gastric cancers [62, 63]. To reveal if the *miR-129* gene is under the regulation of the BRCA1/NEAT1 axis, qRT-PCR assays were performed on MCF10A and MCF10DCIS cells with single or double knockdown of BRCA1 and NEAT1. Consistent with the PCR array data, NEAT1 knockdown led to the induction of miR-129-5p expression in both MCF10A and MCF10DCIS cells, whereas BRCA1 knockdown suppressed its expression (Figure 4B). When BRCA1 and NEAT1 were both knocked down, miR- 129- 5p expression was rescued (Figure 4B). Based on the prior finding that NEAT1 is the downstream of BRCA1, this result suggests that inhibition of miR-129-5p expression by BRCA1 knockdown is NEAT1-dependent. Additionally, BRCA1 overexpression consistently induced miR-129-5p expression in both MCF10A and MCF10DCIS cell lines (Figure 4C). These data together demonstrate that the *miR-129* gene is the downstream of the BRCA1/NEAT1 axis.

Given that the *miR-129* gene is epigenetically silenced in breast and gastric cancers via DNA methylation [62, 63], we hypothesized that NEAT1 may regulate the DNA methylation status of the *miR-129* gene to modulate its expression. To test this hypothesis, we performed bisulfite sequencing analysis of the CpG island of the *miR-129* gene in NEAT1-knockdown MCF10DCIS cells compared with control siRNA-transfected cells. As predicted, NEAT1 knockdown resulted in decreased DNA methylation of the *miR-129* CpG island (66.3 ± 2.6% vs. 88.1 ± 2.8% of the control, *p* < 0.001; *n* = 5) (Figure 4D). This result reveals that NEAT1 epigenetically inhibits miR-129 expression.

### The NEAT1/miR-129-5p signaling axis contributes to enhanced malignant phenotypes and stemness of BRCA1-deficient breast tumor cells

**Figure 5:**
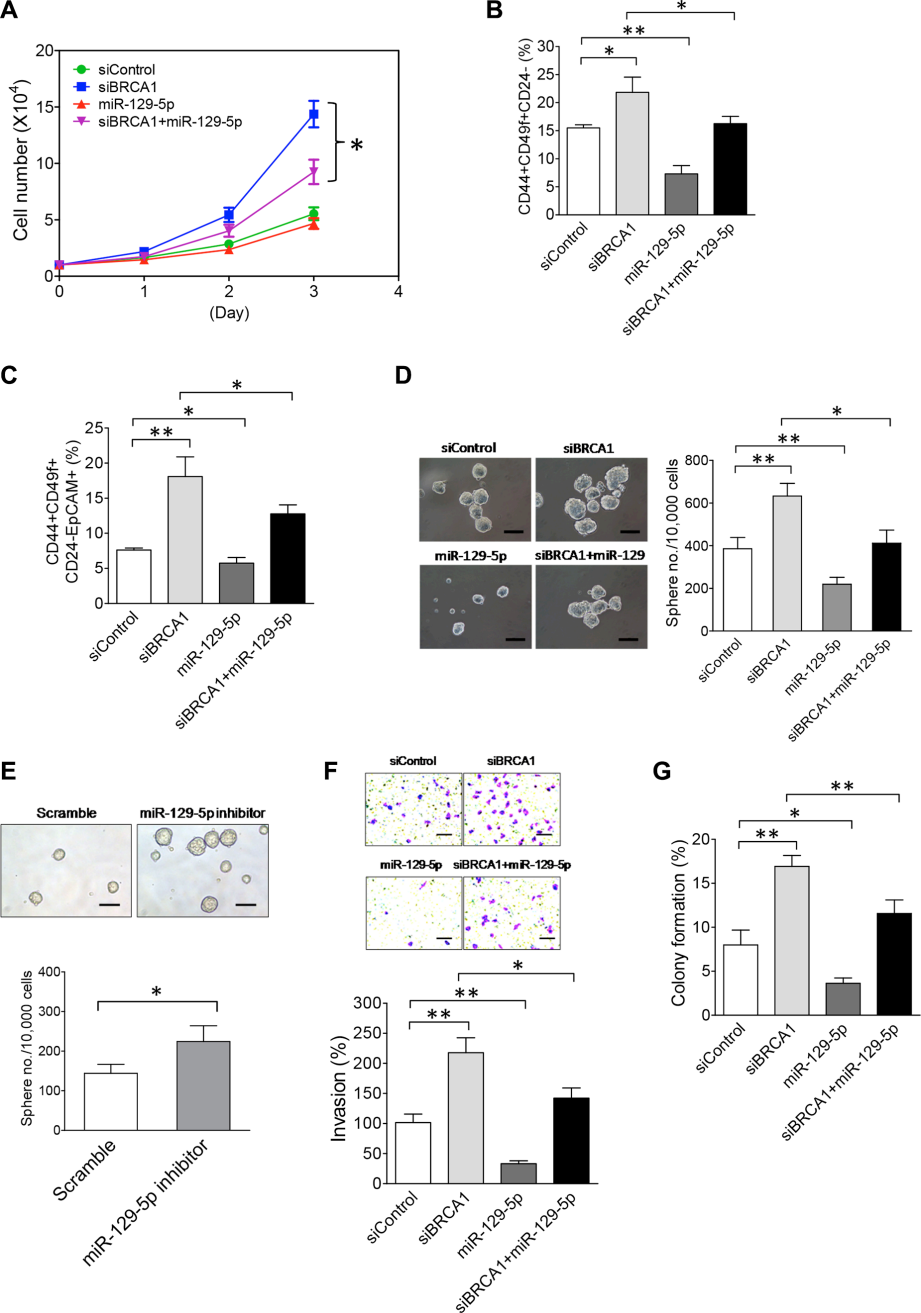
The NEAT1/miR-129-5p axis mediates the effect of BRCA1 deficiency to enhance malignancies and stemness of breast tumor cells (**A**) The miR-129-5p mimic attenuates enhanced proliferation of BRCA1-knockdown MCF10DCIS cells. MCF10DCIS cells were transfected with the control siRNA, BRCA1 siRNA, miR-129-5p mimic or BRCA1 siRNA plus miR- 129- 5p mimic. At 24 hrs after transfection, 10,000 transfected MCF10DCIS cells were seeded for cell proliferation assays as described in Figure 3B. (**B**) The miR-129-5p mimic abolishes the BRCA1-knockdown-induced increase of the BCSC cell population in MCF10DCIS cells. Flow cytometry analysis of BCSC protein antigens CD44, CD49f and CD24 was performed on transfected MCF10DCIS cells 72 hours posttransfection as indicated. The gating and analysis were performed as described in Figure 3C. The quantitative bar graph was plotted based on percentages of CD44+CD49f+CD24– cell subsets from three independent flow cytometry experiments. (**C**) The miR-129-5p mimic reduces the BRCA1-deficiency-induced expansion of the EpCAM+BCSC population in MCF10DCIS cells. Flow cytometry analysis of BCSC protein antigens CD44, CD49f, CD24 and EpCAM was performed on transfected MCF10DCIS cells 72 hours posttransfection as indicated. The gating and analysis were performed as described in Figure 3D. Quantitative analysis of the EpCAM+BCSC subset (CD44+CD49f+CD24–EpCAM+) for a representative flow cytometry experiment is also shown in Supplementary Figure S9. The quantitative bar graph for the measurement of the CD44+CD49f+CD24–EpCAM+ cell subset was plotted based on three independent experiments. (**D**) The miR-129-5p mimic impairs BRCA1-knockdown-induced increases in the size and formation efficiency of BCSC spheres developed from MCF10DCIS cells. At 48 hrs after transfection, 10,000 transfected MCF10DCIS cells as indicated were seeded for stem-cell sphere formation assays. The pictures of formed BCSC spheres are shown in the left panel and the BCSC sphere formation efficiency data are shown in the right panel. The scale bar in sphere pictures indicates 100 μm. (**E**) Inhibition of miR-129-5p enhances the stemness of MCF10A cells. Stem-cell sphere formation assays were performed on MCF10A cells transfected with either the control scramble or miR-129-5p inhibitor RNA. The mammosphere pictures are shown in the top panel and the quantitative bar graph of the mammosphere formation efficiency data is shown in the bottom panel. The scale bar indicates 100 μm. (**F**) The miR-129-5p mimic impairs the BRCA1-knockdown-induced enhancement of invasiveness of MCF10DCIS tumor cells. Transfected MCF10DCIS cells as indicated were subjected to invasion assays 48 hours posttransfection. The stained pictures are shown in the top panel and the quantitative bar graph of invasion data is shown in the bottom panel. The scale bar indicates 100 μm. (**G**) The miR-129-5p mimic attenuates a BRCA1-knockdown-induced increase in anchorage-independent growth of MCF10DCIS tumor cells. Transfected MCF10DCIS cells as indicated were subjected to soft agar assays 48 hours posttransfection. Colony formation efficiency data from three independent experiments were plotted into a bar graph. The error bar in bar graphs represents the SD of the dataset (*n* = 3). *p < 0.05, **p < 0.01.

To reveal the functional role of miR-129-5p in the BRCA1-deficiency-induced malignant phenotypes of breast tumor cells, we performed a miR-129-5p rescue study by co-transfecting the miR-129-5p mimic with BRCA1 siRNA into MCF10DCIS cells. Although ectopic expression of miR-129-5p alone had no significant impact on cell growth, co-expression of miR-129-5p in BRCA1-knockdown MCF10DCIS cells suppressed approximately 55% of increased cell growth (Figure 5A). This rescue study indicates that downregulation of miR-129-5p in BRCA1-knockdown cells contributes to the increased cell growth phenotype.

By using the same miR-129-5p rescue method, we next performed flow cytometry analysis to examine the effect of miR-129-5p on the BCSC population. As shown in Figure 5B and 5C, ectopic expression of miR-129-5p alone significantly reduced both CD44+CD49f+CD24– (7.31 ± 1.49% vs. 15.51 ± 0.55% of the control, *p* < 0.01; *n* = 3) and CD44+CD49f+CD24–EpCAM+ BCSC subsets (5.74 ± 0.81% vs. 7.62 ± 0.28% of the control, *p* < 0.05; *n* = 3), indicating that miR-129-5p is an intrinsic suppressor of BCSCs. Co-transfection of miR-129-5p with BRCA1 siRNA partially abrogated the expanded CD44+CD49f+CD24– (from 21.85 ± 2.71% down to 16.26 ± 1.29%, *p* < 0.05; *n* = 3) and CD44+CD49f+CD24–EpCAM+ BCSC subsets (from 18.07 ± 2.83% down to 12.77 ± 1.27%, *p* < 0.05; *n* = 3) induced by BRCA1 knockdown (Figure 5B, 5C). We verified these findings using stem-cell sphere formation assays. Ectopic expression of miR-129-5p alone attenuated self-renewal (indicated by the reduced sphere number) and the proliferation rate (indicated by the smaller sphere size) of BCSCs in MCF10DCIS (Figure 5D). When co-transfected with siBRCA1, the miR-129-5p mimic significantly inhibited the increased self-renewal and proliferation of BCSCs induced by BRCA1-knockdown (Figure 5D).

To understand whether inhibition of miR-129-5p has an opposite effect to promote self-renewal of breast stem cells, we transfected the miR-129-5p inhibitor RNA (antagomir) into MCF10A cells that express normal levels of miR-129-5p and performed sphere formation assays to examine the effect. Indeed, inhibition of miR- 129-5p promoted self-renewal of breast stem cells in MCF10A cells when compared with the scramble control (Figure 5E). Moreover, the transfection of the miR-129- 5p mimic alone suppressed invasiveness and anchorage-independent growth of MCF10DCIS cells and its co- transfection with siBRCA1 impaired the enhancing effects of BRCA1 knockdown on these two malignant phenotypes (Figure 5F, 5G). These findings demonstrate that downregulation of miR-129-5p expression by upregulated NEAT1 contributes to enhanced cell proliferation, stemness, invasiveness and anchorage-independent growth of BRCA1-deficient breast tumor cells.

### WNT4 is a target of miR-129-5p and downstream of the BRCA1/NEAT1 axis

**Figure 6:**
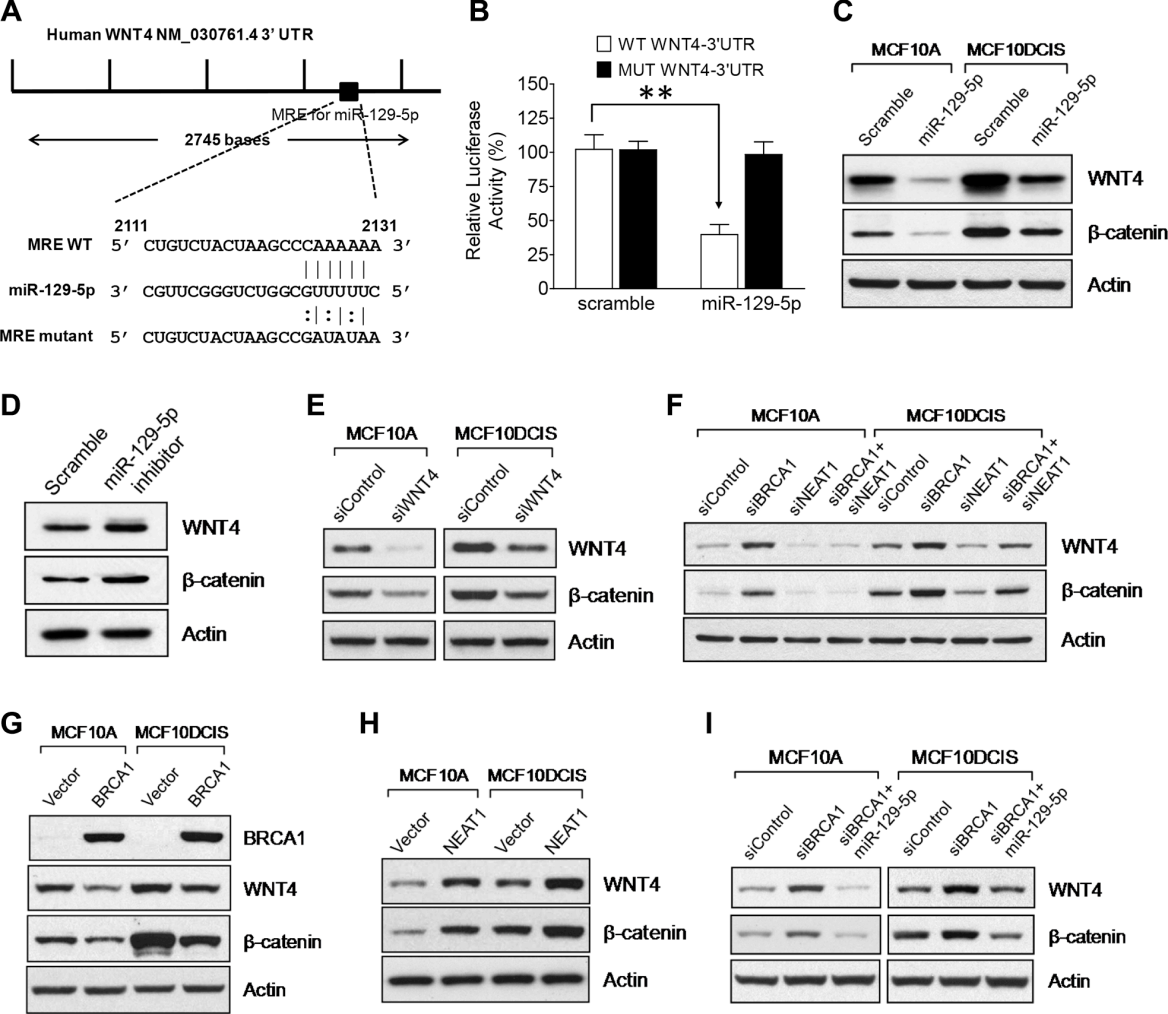
*WNT4* is a miR-129-5p target gene that is regulated by the BRCA1/NEAT1/miR-129-5p axis (**A**) A map for the predicted miR-129-5p targeting site in the 3′-untranslated region of the WNT4 mRNA. A DNA fragment with mutations in the seeding site of WNT4 3′-UTR was used to construct the mutant reporter and its RNA sequence is shown under the map with its wild-type and miR-129- 5p sequences. (**B**) The miR-129-5p mimic inhibits the luciferase expression of the wild-type, but not the mutated WNT4 3′- UTR reporter. HEK-293T cells were transfected with the wild-type or mutated WNT4 3′-UTR reporter plasmid DNA along with either the control scramble dsRNA or the miR-129-5p mimic. All cell samples were also co-transfected with Renilla plasmid DNA, which was used as a transfection efficiency control. Dual Luciferase assays were performed on transfected cells 24 hrs posttransfection. The measured Luciferase activity values were normalized by Renilla activity values. The error bar represents the SD of the dataset (*n* = 3). **p < 0.01. (**C**) The miR-129-5p mimic downregulates WNT4 expression and WNT signaling activity. Western blot analysis of WNT4, β-catenin and β-actin was performed on scramble dsRNA-tansfected and miR-129-5p-transfected MCF10A and MCF10DCIS cells. (**D**) Inhibition of miR-129-5p in MCF10A cells leads to WNT4 upregulation and activation of WNT signaling. 48 hours posttransfection, Western blot analysis of WNT4, β-catenin and β-actin was performed on MCF10A cells transfected with either the scramble or miR-129-5p inhibitor RNA. (**E**) WNT4 is functionally required for endogenous WNT signaling activity. Western blot analysis of WNT4, β-catenin and β-actin was performed on MCF10A and MCF10DCIS cells transfected with the control siRNA or the WNT4 siRNA for 48 hrs. (**F**) Upregulation of WNT4 expression and activation of WNT signaling by BRCA1 knockdown are NEAT1-dependent. Western blot analysis of WNT4, β-catenin and β-actin was performed on MCF10A and MCF10DCIS cells transfected with the control siRNA, the siRNA targeting BRCA1 or NEAT1, or the siRNA combination targeting both genes for 48 hours. (**G**) Ectopic expression of BRCA1 downregulates WNT4 expression and suppresses WNT signaling activity. Western blot analysis of BRCA1, WNT4, β-catenin and β-actin was performed on MCF10A and MCF10DCIS cells transfected with the empty vector or BRCA1 expression plasmid DNA. (**H**) Ectopic expression of NEAT1 upregulates WNT4 expression and activates WNT signaling activity. Western blot analysis of WNT4, β-catenin and β-actin was performed on MCF10A and MCF10DCIS cells transfected with the empty vector or NEAT1 expression plasmid DNA. (**I**) Upregulation of WNT4 expression and activation of WNT signaling by BRCA1 knockdown are abolished by the miR-129-5p mimic. Western blot analysis of WNT4, β-catenin and β-actin was performed on MCF10A and MCF10DCIS cells transfected with the control siRNA, the BRCA1 siRNA or BRCA1 siRNA plus miR-129-5p mimic for 48 hours. β-actin was used as a protein loading control for all Western blot analyses shown here.

To unravel how the NEAT1/miR-129-5p axis contributes to enhanced malignancies caused by BRCA1 deficiency, we searched the putative gene targets of miR-129-5p using PicTar, TargetScan, and Miranda algorithms [64–66]. Among the putative targets, we identified WNT4 (Figure 6A), which has been reported to be involved in mammary stem cell regulation [67]. To verify that WNT4 is a target of miR-129-5p, we performed luciferase reporter analysis of the WNT4 3′-untranslated region (3′-UTR). Co-transfection of miR- 129-5p with the wild-type WNT4 3′-UTR reporter led to approximately 60% suppression of the reporter activity (*p* < 0.01, *n* = 3), whereas miR-129- 5p had no effect on the activity of the mutated WNT4 3′-UTR reporter with mutations at the miR-129-5p recognition site (Figure 6B). We also conducted Western blot experiments to examine WNT4 expression in miR- 129-5p-overexpressing MCF10A and MCF10DCIS cells compared to control scramble RNA-transfected cells. Consistent with the reporter data, miR- 129- 5p overexpression significantly suppressed WNT4 expression in both cell lines (Figure 6C), demonstrating that WNT4 is the genuine target of miR-129-5p. We also examined whether WNT signaling activity (indicated by β-catenin stabilization) correlates with WNT4 expression status. Indeed, as shown in Figure 6C, β-catenin protein levels positively correlated with WNT4 protein levels and miR-129-5p overexpression concurrently downregulated both WNT4 and β-catenin protein levels.

To further confirm the result of the miR-129-5p mimic, we transfected the miR-129-5p inhibitor RNA into MCF10A cells with normal expression levels of miR-129-5p and examined the effect of miR-129-5p inhibition on WNT4 and β-catenin protein levels. As expected, inactivation of miR-129-5p resulted in elevated protein levels of WNT4 and β-catenin in MCF10A cells (Figure 6D). To reveal if WNT4 is an activator of WNT signaling in both MCF10A and MCF10DCIS cells, we performed WNT4 knockdown and Western blot studies. The results convincingly show the positive role of WNT4 in WNT signaling in both cell lines (Figure 6E). These data together indicate that miR-129-5p inhibits WNT signaling via downregulation of WNT4 expression.

To test if WNT4 is downstream of the BRCA1/NEAT1 axis, we performed Western blot analysis to examine WNT4 expression in MCF10A and MCF10DCIS cells with single knockdown of either BRCA1 or NEAT1, or with double knockdown of both genes. BRCA1 knockdown resulted in upregulation of WNT4 expression and increased stabilization of β-catenin, whereas NEAT1 knockdown caused an opposite outcome (Figure 6F). These results indicate that BRCA1 and NEAT1 are a suppressor and activator of WNT4 expression and WNT signaling, respectively. Co-knockdown of NEAT1 blocked the BRCA1-knockdown-induced upregulation of WNT4 and β-catenin in both MCF10A and MCF10DCIS cells (Figure 6F), indicating that induction of WNT4 expression and WNT signaling activity by BRCA1 deficiency is NEAT1-dependent. In contrast, ectopic overexpression of BRCA1 downregulated WNT4 and β-catenin protein levels in both MCF10A and MCF10DCIS (Figure 6G). To further confirm the role of NEAT1 in the regulation of the miR-129-5p/WNT4 axis, NEAT1 overexpression experiments were performed. As shown in Figure 6H, Western blot analyses showed that NEAT1 overexpression resulted in increased WNT4 and β-catenin protein levels in both MCF10A and MCF10DCIS cells, indicating that NEAT1 is an activator for WNT4 expression and WNT signaling.

To reveal if the NEAT1/miR-129-5p signaling axis mediates BRCA1-deficiency-induced upregulation of WNT4 expression and WNT signaling, Western blot analysis of WNT4 and β-catenin in BRCA1-knockdown cells with or without co-overexpression of miR- 129- 5p was performed. Indeed, in both MCF10A and MCF10DCIS cells co-overexpression of miR-129-5p abolished induction of WNT4 expression and activation of WNT signaling induced by BRCA1 knockdown (Figure 6I). These findings, taken together, indicate that WNT4 is downstream of the BRCA1/NEAT1/miR-129-5p signaling axis.

### WNT4 is functionally involved in enhanced malignant phenotypes and stemness of breast tumor cells with BRCA1 deficiency

**Figure 7:**
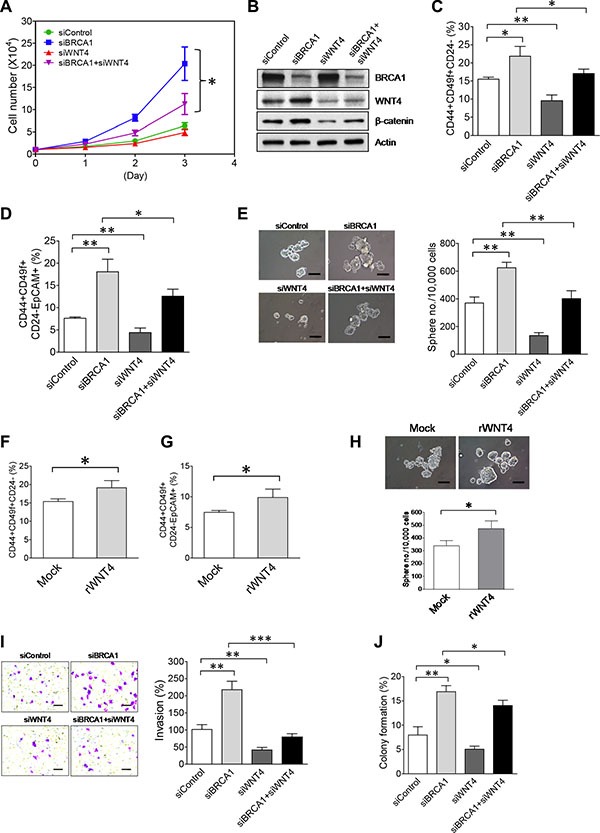
Upregulation of WNT4 by the NEAT1-miR129 axis is functionally implicated in promoting malignant phenotypes and stemness of BRCA1-deficient breast tumor cells (**A**) WNT4 knockdown attenuates enhanced proliferation of BRCA1-knockdown MCF10DCIS. MCF10DCIS cells were transfected with the control siRNA, BRCA1 siRNA, WNT4 siRNA, or combined siRNAs targeting both BRCA1 and WNT4. At 24 hrs after transfection, 10,000 siRNA-transfected MCF10DCIS cells were seeded for cell proliferation assays as described in Figure 3B. (**B**) Co-knockdown of WNT4 abolishes the BRCA1-knockdown-induced activation of WNT signaling in MCF10DCIS cells. Western blot analysis of BRCA1, WNT4, β-catenin and β-actin was performed on siRNA-transfected MCF10DCIS cells 48 hours posttransfection as indicated. (**C**) Co-knockdown of WNT4 abolishes the BRCA1-knockdown-induced increase of the BCSC cell population in MCF10DCIS cells. Flow cytometry analysis of three BCSC protein markers (CD44, CD49f and CD24) was performed on siRNA-transfected MCF10DCIS cells 72 hours posttransfection as indicated. The quantitative bar graph for the measurement of the CD44+CD49f+CD24– cell subset in siRNA-transfected MCF10DCIS cells was plotted based on three independent experiments as described in Figure 3C. (**D**) Co-knockdown of WNT4 attenuates the BRCA1-knockdown-induced expansion of the EpCAM+BCSC population in MCF10DCIS cells. Flow cytometry analysis of four BCSC protein markers (CD44, CD49f, CD24 and EpCAM) was performed on siRNA-transfected MCF10DCIS cells 72 hours posttransfection as indicated. The quantitative bar graph for the measurement of the CD44+CD49f+CD24–EpCAM+ cell subset in siRNA-transfected MCF10DCIS cells was plotted based on three independent experiments as described in Figure 3D. Quantitative analysis of the EpCAM+BCSC subset (CD44+CD49f+CD24–EpCAM+) for a representative flow cytometry experiment is also shown in Supplementary Figure S10. (**E**) Co-knockdown of WNT4 impairs BRCA1-knockdown-induced increases in the size and formation efficiency of BCSC spheres generated from MCF10DCIS cells. At 48 hrs after transfection, 10,000 siRNA-transfected MCF10DCIS cells as indicated were seeded for stem-cell sphere formation assays. The pictures of formed spheres are shown in the left panel and the sphere formation efficiency data are shown in the right panel. (**F**) Treatment with the recombinant WNT4 cytokine induces the increase of BCSCs in MCF10DCIS cells. After treatment with recombinant WNT4 (50 nM) for 72 hrs, treated MCF10DCIS cells were subjected to the same flow cytometry analysis as described in (C). (**G**) Treatment with the recombinant WNT4 cytokine enhances the expansion of EpCAM+BCSCs in MCF10DCIS cells. Flow cytometry analysis of EpCAM+BCSCs in rWNT4-treated MCF10DCIS cells was performed as described in (D). (**H**) Treatment with the recombinant WNT4 cytokine enhances self-renewal of BCSCs in MCF10DCIS cells. After treatment with recombinant WNT4 (rWNT4, 50 nM) for 72 hrs, 10,000 treated MCF10DCIS cells were subjected to stem-cell sphere formation assays. During sphere formation, the sphere culture medium was supplemented with rWNT4 (50 nM). The pictures of formed BCSC spheres are shown in the top panel and the sphere formation efficiency data are shown in the bottom panel. (**I**) Co-knockdown of WNT4 abolishes the BRCA1-knockdown-enhanced invasiveness of MCF10DCIS tumor cells. SiRNA-transfected MCF10DCIS cells as indicated were subjected to invasion assays 48 hours posttransfection. The stained pictures are shown in the left panel and the quantitative bar graph of invasion data is shown in the right panel. (**J**) Co-knockdown of WNT4 moderately impairs the BRCA1-knockdown-promoted anchorage-independent growth of MCF10DCIS tumor cells. SiRNA-transfected MCF10DCIS cells as indicated were subjected to soft agar assays 48 hours posttransfection. Colony formation efficiency data from three independent experiments were plotted into a bar graph. The scale bar shown in sphere and invasion pictures (E, H, I) indicates 100 μm. The error bar represents the SD of the dataset (*n* = 3). *p < 0.05, **p < 0.01, ***p < 0.001.

As our aforementioned findings (Figure 6) indicate that WNT4 is downstream of the BRCA1/NEAT1/miR-129-5p axis and activates oncogenic WNT signaling in breast tumor cells, we postulated that WNT4 is involved in enhancing malignant phenotypes and stemness of BRCA1-deficient breast tumor cells. To test this hypothesis, we performed experiments of single and double knockdown of WNT4 and BRCA1. As shown in Figure 7A, co- knockdown of WNT4 by a siRNA [68] substantially inhibited the increased proliferation (∼60% reduction) of BRCA1-knockdown MCF10DCIS cells. This result indicates that WNT4 is required for the increased cell proliferation of BRCA1-deficient MCF10DCIS cells. Consistently, co-knockdown of WNT4 abolished the BRCA1-knockdown-induced activation of WNT signaling in MCF10DCIS cells (Figure 7B).

To unveil the functional role of WNT4 in BCSCs, we performed flow cytometry analysis of BCSCs in MCF10DCIS cells with single and double knockdown of WNT4 and BRCA1. Knockdown of WNT4 in MCF10DCIS cells caused a significant reduction in both CD44+CD49f+CD24– (9.55 ± 1.59% vs. 15.51 ± 0.55% of the control, *p* < 0.01; *n* = 3) and CD44+CD49f+CD24–EpCAM+ BCSC subsets (4.39 ± 1.03% vs. 7.62 ± 0.28% of the control, *p* < 0.01; *n* = 3) (Figure 7C, 7D). In the co-knockdown experiment, WNT4 knockdown suppressed over 50% of the increase in the CD44+CD49f+CD24– (from 21.85 ± 2.71% down to 17.06 ± 1.22% relative to 15.51 ± 0.55% of the control, *p* < 0.05; *n* = 3) and CD44+CD49f+CD24–EpCAM+ (18.07 ± 2.83% down to 12.59 ± 1.56% relative to 7.62 ± 0.28% of the control, *p* < 0.05; *n* = 3) BCSC subsets induced by BRCA1 knockdown (Figure 7C, 7D). Consistent with flow cytometry results, stem-cell sphere formation studies indicate that WNT4 is intrinsically required for self-renewal of BCSCs, and also for the increased stemness caused by BRCA1 deficiency (Figure 7E).

Given that WNT4 is a secreted signaling molecule, we therefore examined whether secreted WNT4 promotes stemness of BCSCs. To test the effect of secreted WNT4 on BCSCs, MCF10DCIS cells were treated with recombinant WNT4 (rWNT4) and analyzed using flow cytometry and stem-cell sphere formation assays. Both studies consistently showed that rWNT4 treatment promoted stemness of MCF10DCIS, indicated by expanded CD44+CD49f+CD24– (19.13 ± 1.94% vs. 15.38 ± 0.73% of the control, *p* < 0.05; *n* = 3) and CD44+CD49f+CD24–EpCAM+ (9.89 ± 1.37% vs. 7.46 ± 0.31% of the control, *p* < 0.05; *n* = 3) BCSC subsets and the increased number of rWNT4-treated BCSC spheres (Figure 7F, 7G and 7H). Moreover, results from invasion and soft agar experiments showed that co-knockdown of WNT4 in BRCA1-knockdown MCF10DCIS cells abolished enhanced invasiveness and moderately impaired promoted anchorage-independent growth (Figure 7I, 7J). These results, taken together, indicate that as a downstream effector of the NEAT1/miR-129-5p axis, upregulated WNT4 is functionally required for enhanced malignant phenotypes and stemness of breast tumor cells induced by BRCA1 abrogation.

### The relevance of the BRCA1/NEAT1/miR-129-5p axis in breast cancer

To reveal how relevant the BRCA1/NEAT1/miR-129-5p signaling axis is to breast cancer, we performed *in silico* analysis of publicly available cancer-related expression databases and published expression data to examine the expression correlation between these three molecules. Due to the lack of available public expression databases and published data gathering mRNA, lncRNA and miRNA expression profiles together, we focused on analysis of breast cancer cell lines that have been profiled for these three types of RNA [69–71]. Moreover, the homogeneity of breast cancer cell lines makes it possible to perform more accurate analysis.

Neve *et al.* profiled mRNA and some lncRNA expression in 51 breast cancer cell lines [70], and the expression information for *BRCA1* and *NEAT1* genes from these datasets was obtained from Oncomine (https://www.oncomine.org) [69]. We obtained miR-129-5p data from a publication by Riaz *et al.,* who profiled the miRNA expression of 51 breast cancer cell lines [71]. Thirty nine cell lines of Neve's datasets overlaps with those of Riaz's dataset. Owing to HCC1937 carrying the mutated *BRCA1* gene, this line was excluded and expression datasets of remaining thirty eight cell lines (Supplementary Table S1) were subjected to *in silico* expression correlation analysis. Linear regression analysis was performed to evaluate the expression correlation coefficiency between two genes and statistical significance of this correlation.

**Figure 8:**
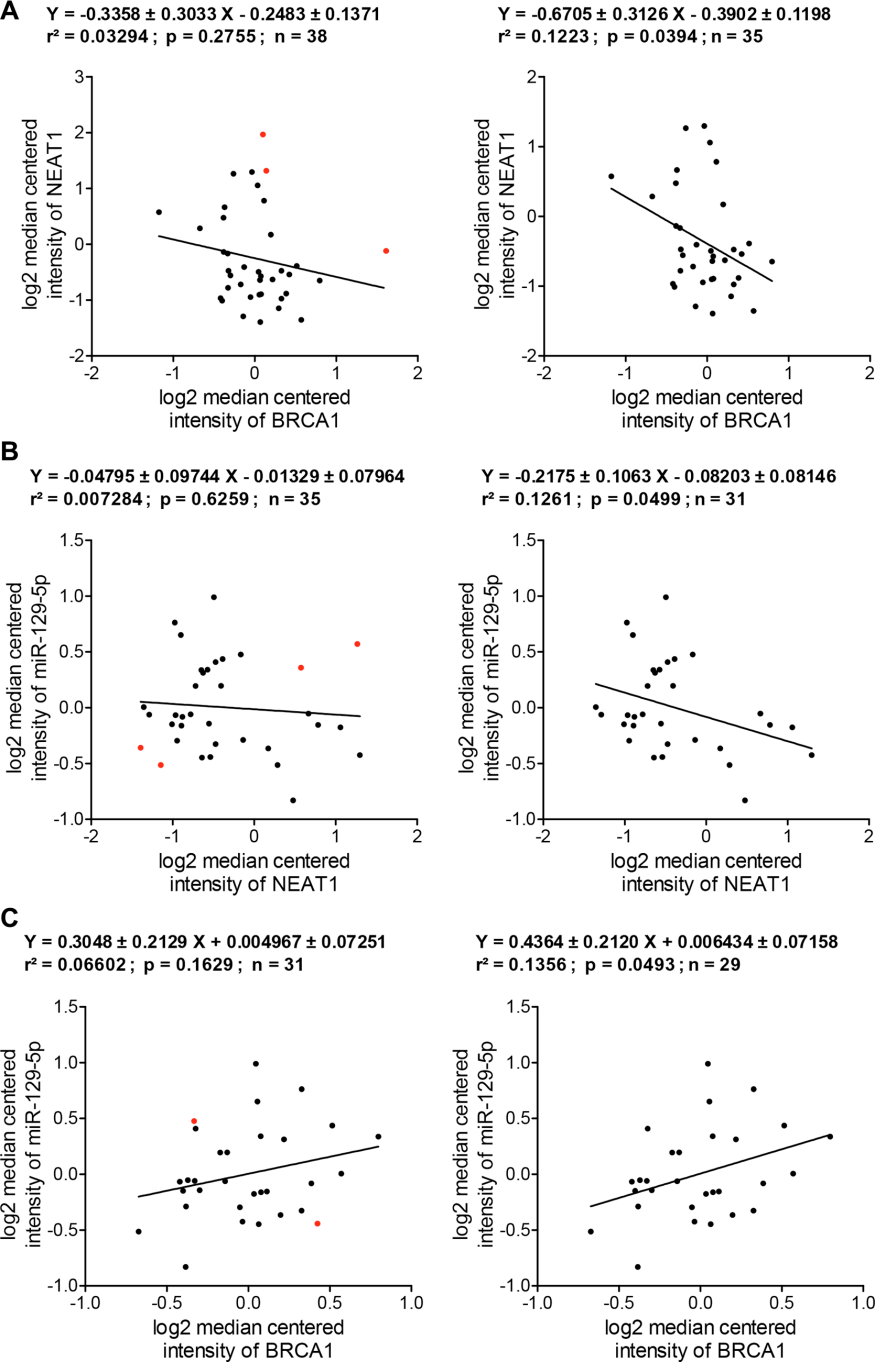
*In silico* expression correlation analysis of BRCA1, NEAT1 and miR-129-5p in a cohort of human breast cancer cell lines (**A**) Regression analysis of the expression correlation between BRCA1 and NEAT1 in breast cancer cell lines. The left regression analysis plot was made according to BRCA1 and NEAT1 expression datasets from 38 BC lines. The right regression analysis plot was made according to the datasets of 35 BC lines after the data of three cell lines (SUM-190PT, SUM-225CWN, and T47D; indicated by red dots shown in the left plot) with a poor correlation were excluded from analysis. (**B**) Regression analysis of the expression correlation between NEAT1 and miR-129-5p in breast cancer cell lines. The left regression analysis plot was made according to NEAT1 and miR- 129-5p expression datasets from 35 BC lines that were narrowed down from analysis shown in the right panel of (A). The right regression analysis plot was made according to the datasets of 31 BC lines after the data of four cell lines (HCC70, MDA-MB-231, SUM-185PE, and SUM-52PE; indicated by red dots shown in the left plot) with a poor correlation were excluded from analysis. (**C**) Regression analysis of the expression correlation between BRCA1 and miR-129-5p in breast cancer cell lines. The left regression analysis plot was made according to BRCA1 and miR-129-5p expression datasets from 31 BC lines that were narrowed down from analysis shown in the right panel of (B). The right regression analysis plot was made according to the datasets of 29 BC lines after the data of two cell lines (BT- 483 and HCC1143; indicated by red dots shown in the left plot) with a poor correlation were excluded from analysis. All of expression correlation analyses shown here were based on expression data values of Supplementary Table S1.

Given variable genetic and epigenetic alterations among these breast cancer cell lines, we predicted that some cell lines had lost the BRCA1/NEAT1/miR-129- 5p regulation axis. Therefore these cell lines would need to be identified and excluded from the analysis to obtain more meaningful analyzed results. Regression analysis of BRCA1 and NEAT1 expression in 38 BC lines showed a negative correlation trend (correlation coefficiency = –0.3358 ± 0.3033) although it was not statistically significant (*p* = 0.2755) (Figure 8A, the left plot). Three cell lines (SUM-190PT, SUM-225CWN, and T47D) were identified to have a poor correlation between BRCA1 and NEAT1 RNA expression (Figure 8A, three red dots in the left plot). After exclusion of these three lines, expression levels of NEAT1 in 35 BC lines negatively correlated with those of BRCA1 (correlation coefficiency = –0.6705 ± 0.31260) in a statistically significant manner (*p* = 0.0394) (Figure 8A, the right plot). By *in silico* analysis of this 38-line cohort, we revealed that over 90% of these BC lines manifested the negative correlation between BRCA1 and NEAT1, consistent with the BRCA1/NEAT1 regulation axis identified from our aforementioned studies.

These 35 cell lines showing the trend of BRCA1/NEAT1 regulation were further subjected to expression correlation analysis of both NEAT1 and miR-129-5p. As shown in Figure 8B (the left plot), the expression correlation between these two non-coding RNAs was poor (*p* = 0.6259) possibly due to loss of NEAT1/miR-129-5p regulation in some cell lines (indicated by red dots). This was expected as miR-129-5p expression is epigenetically regulated by NEAT1 and any other epigenetic alterations may interfere with this regulatory axis. After removal of the four cell lines (HCC70, MDA-MB-231, SUM-185PE, and SUM-52PE) with a poor correlation, the remaining 31 cell lines displayed a negative correlation between NEAT1 and miR-129-5p expression (correlation coefficiency = –0.2175 ± 0.1063) in a statistically significant manner (*p* = 0.0499) (Figure 8B, the right plot), consistent with our finding that NEAT1 negatively regulates miR-129-5p expression.

We further analyzed the expression correlation between BRCA1 and miR-129-5p in these 31 BC lines. BRCA1 mRNA expression levels tended to positively correlate with miR-129-5p levels (correlation coefficiency = 0.3048 ± 0.2129) although it was not statistically significant (*p* = 0.1629) (Figure 8C, the left plot). After exclusion of two cell lines (BT-483 and HCC1143, indicated by red dots in the left plot of Figure 8C) identified to have a poor correlation, the remaining 29 BC lines showed a positive correlation between BRCA1 and miR-129-5p (correlation coefficiency = 0.4364 ± 0.2120) in a statistically significant manner (*p* = 0.0493) (Figure 8C, the right plot). Without these sequential analyses, there is no correlation between BRCA1 and miR-129-5p (correlation coefficiency = –0.02839 ± 0.1607; *p* = 0.8608; *r*^2^ = 0.000866) from analysis of all 38 cell lines. This indicates that the exclusion of uncorrelated cell lines is important for obtaining the meaningful expression correlation data. These *in silico* analyses, taken together, indicate that 29 (76.3%) out of 38 BC lines exhibited the trend of BRCA1/NEAT1/miR-129-5p axis regulation. Therefore, our findings suggest that this identified signal axis is potentially relevant in the significant portion of breast cancers.

## DISCUSSION

**Figure 9:**
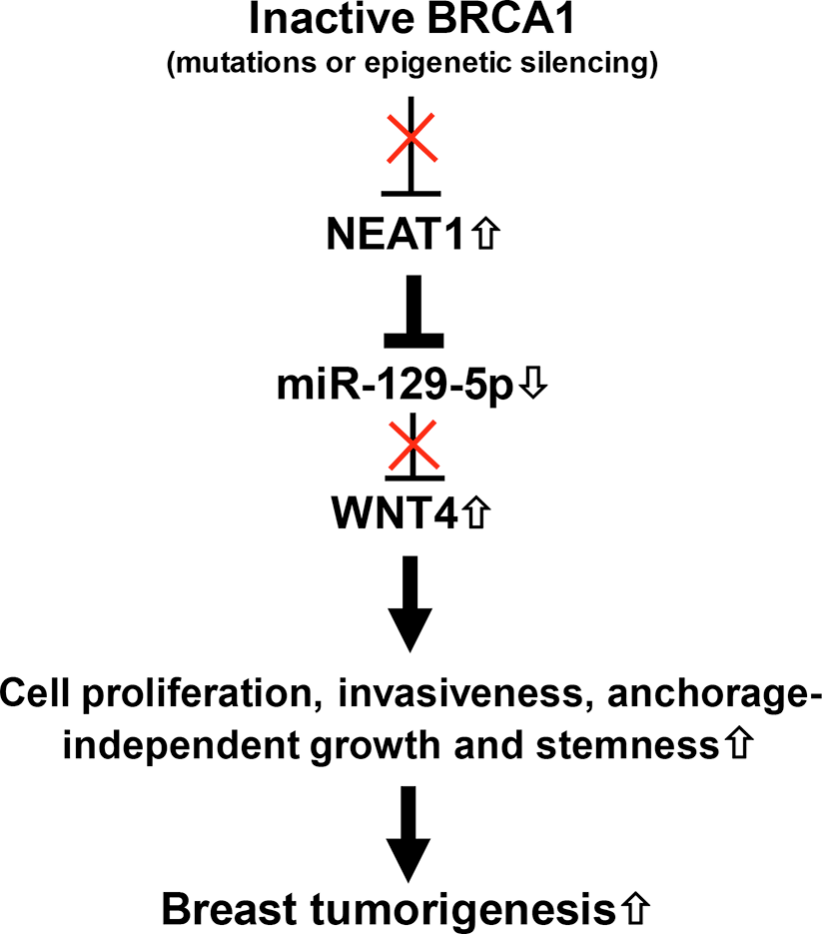
The model for the role of the BRCA1/NEAT1/miR-129-5p/WNT4 signaling axis in BRCA1-deficiency-driven breast tumorigenesis

In this study, we report that lncRNA NEAT1 expression is negatively regulated by BRCA1, potentially through the binding of BRCA1 to its cognate binding site upstream of the *NEAT1* gene. To our knowledge, this study is the first report that expression of the lncRNA can be regulated by BRCA1. Moreover, NEAT1 is crucial for tumorigenicity of BRCA1-deficient breast cancer. Through our mechanistic studies we have identified the NEAT1/miR-129-5p/WNT4 axis and revealed that dysregulation of this signaling axis contributes to BRCA1-deficiency-induced malignant phenotypes in breast cancer cells, such as increases in cell proliferation, invasiveness, anchorage-independent growth and stemness. Moreover, our *in silico* correlation analysis indicates that a significant portion of breast cancer cell lines (> 70%) manifested the regulation trend of the BRCA1/NEAT1/miR-129-5p axis. According to our findings, we propose a model wherein after BRCA1 function is downregulated or inactivated by gene mutations or epigenetic silencing (e.g. DNA methylation), oncogenic NEAT1 is upregulated to epigenetically downregulate tumor-suppressive miR-129-5p expression and subsequently activate WNT4 expression. These sequential alterations promote breast tumorigenesis by increasing stemness and enhancing malignant phenotypes (Figure 9).

It has been shown that the CD49f+EpCAM+ luminal progenitor subpopulation was significantly expanded in *BRCA1* mutant breast tissues compared with age-matched normal counterparts [50, 52]. Our finding showed that BRCA1 knockdown induced the expansion of the CD49f+EpCAM+ subpopulation in the CD44+CD24– cell subset of MCF10DCIS tumor cells, confirming the relationship between BRCA1 deficiency and luminal progenitors [50, 52]. It has been known that as a BL-DCIS tumor cell model, MCF10DCIS cells contain bipotent BCSCs [72]. Given that BRCA1 deficiency has been shown to block the differentiation of mammary stem/progenitor cells and expand their populations [50–53], our finding suggests that bipotent BCSCs in MCF10DCIS cells express luminal stem/progenitor cell marker EpCAM in addition to the basal stem/progenitor CD44+CD49f+CD24– profile and BRCA1 knockdown can expand this EpCAM+CSC population by blocking CSC differentiation.

Importantly, we have identified NEAT1 as a novel, key BCSC regulator that is required for BRCA1-deficiency-induced BCSC expansion. Furthermore, we have revealed that NEAT1 upregulation by BRCA1 deficiency results in activating the expression of the stem-cell factor WNT4 by suppressing miR-129-5p expression. Our functional studies indicate that WNT4 upregulation contributes to the expansion of BCSCs. Given that WNT4 upregulation activates WNT signaling, this suggests that activated WNT signaling potentially mediates the effect of WNT4 on promoting BCSC generation in BRCA1-deficient breast tumors. The discovery of the BRCA1/NEAT1/miR-129-5p/WNT4 axis and the pivotal roles of WNT4 in WNT signaling activation as well as in the enhancement of BCSC generation suggest that WNT signaling is a potential therapeutic target for breast cancer with alterations in this signaling axis.

Our findings reveal that NEAT1 epigenetically regulates the expression of the microRNA gene. We found that NEAT1 inhibited miR-129 expression by increasing the DNA hypermethylation of the CpG island in the *miR-129* gene. To our knowledge, it is the first time that NEAT1 has been shown to modulate the DNA methylation status of a microRNA gene. It is known that the DNA methylation status of genes can be regulated by both directions of DNA methylation (methylation vs. demethylation), which are mediated by epigenetic enzymes (e.g. DNA methyltransferases for DNA methylation; AID/Apobec and TET enzymes for DNA demethylation). Therefore, future work will be needed to reveal whether this epigenetic regulation is achieved by binding of NEAT1 to epigenetic regulators that modulate DNA methylation and demethylation, NEAT1-triggered changes in epigenetic modulator gene expression, or both mechanisms.

In conclusion, for the first time we have identified that NEAT1 plays oncogenic roles in promoting tumorigenicity and stemness of BRCA1-deficient breast cancer. This NEAT1-dependent oncogenic mechanism involves the stem-cell-regulatory factor WNT4, which contributes to facilitating the generation of BCSCs. The NEAT1-mediated regulatory network may have a broad implication to other tumorigenic mechanisms involving NEAT1 dysregulation. Therefore, our findings highlight the critical nature of non-coding RNA-regulatory networks in the regulation of CSC generation and tumorigenicity, and the necessity of further investigation.

## MATERIALS AND METHODS

### Cell culture

The human immortalized breast epithelial cell line MCF10A and breast cancer cell lines MCF7 and HCC1937 were purchased from the American Type Culture Collection (ATCC, Manassas, VA, USA). The human DCIS cell line MCF10DCIS.COM (MCF10DCIS) was purchased from Asterand USA (Detroit, MI, USA). These cell lines were cultured according to manufacturer's instructions.

### Mice

Wild-type and *Brca1* mutant mammary glands used in qRT-PCR and *in situ* hybridization studies were isolated from *C57BL/6* and *MMTV-cre;Brca1*^co/co^ mice. *MMTV-cre;Brca1*^co/co^ mice carried two *Brca1* conditional alleles (*Brca1*^Co/Co^) whose knockout was driven by *MMTV-cre* [44, 73]. The cre-mediated knockout of *Brca1*^Co^ alleles generates mutated *Brca1* alleles with the deletion of exon 11. *MMTV-cre;Brca1*^co/co^ mice have long tumor latency and need over a one-year to a two-year period to develop mammary tumors (20–30% tumor formation rate) [73].

### siRNA, miR-129-5p mimic and inhibitor transfections

siRNA, miR-129-5p mimic and inhibitor transfections were performed with 20 nM of each reagent using OligofectamineTM RNAiMAX (Invitrogen, Carlsbad, CA, USA) according to the manufacturer's instructions. The miR-129-5p mimic and inhibitor were obtained from Sigma (St.Louis, MO, USA). The siRNA sequences used in the study are: scramble, 5′-UAACUCGCUCGAAGGAAUC-3′; siBRCA1-1, 5′-CAGCAGUUUAUUACUCACU-3′ [42]; siBRCA1-2, 5′-ACCAUACAGCUUCAUAAAU-3′ [43]; siNEAT1-1, 5′-UGGUAAUGGUGGAGGAAGA-3 [31]; siNEAT1-2, 5′-GUGAGAAGUUGCUUAGAAA-3′ [31]; siWNT4, 5′- GGAUGCUCUGACAACAUCG-3′ [68].

### Transwell migration and invasion assays

Transwell migration assays were carried out as previously described [54]. Briefly, 2.5 × 10^4^ cells were seeded in the upper transwell chamber insert for the migration assay. For the invasion assay, 40 μl of matrigel was added into the upper transwell chamber insert to form a thin gel layer in a 37°C incubator for 15–30 minutes and 2.0 × 10^5^ cells were then seeded on the top. The lower chamber was filled with the complete culture medium containing 10% serum. Cells were allowed to migrate or invade towards the serum gradient for 24 hours. Migrated or invaded cells were stained with 1% crystal violet and counted using a phase-contrast microscope. Five random fields were counted per experiment.

### Soft agar assays

To perform soft agar assays, the bottom agar (0.6% agar) was prepared by mixing 1.2% agar (it was pre-warmed at 40°C before mixing) with the 2× complete culture medium at the 1:1 ratio and 2 ml of the prepared bottom agar was plated into each well of the six-well plate. Once the bottom agar was solidified, the top agar (0.3% agar) was prepared by mixing 0.6% agar (it was pre-warmed at 40°C before mixing) with the 2× complete culture medium at the 1:1 ratio. 2.0 × 10^4^ cells were immediately suspended in 1 ml of the top agar and plated into six-well plates. After the top agar became solidified, 2 ml of the culture medium was added and soft agar plates were maintained in a cell culture incubator until spheroid colonies formed. Formed colonies in agar were stained with 0.005% crystal violet and counted (≥ 50 μm) under a dissection microscope. To calculate the colony formation efficiency, the number of formed colonies was divided by the total number of seeded cells.

### Tumorigenicity assay

1 × 10^6^ of puromycin-selected scramble shRNA-expressing (as a control) or shNeat1-expressing tumor cells that were isolated from Brca1-deficient mammary tumors developed in *MMTV-cre;Brca1*^Co/Co^ mice were injected into the fourth mammary fat pad of syngeneic female mice with age of 6 weeks (*n* = 6 for each transfectant). The length and width of tumors were measured weekly with a caliber to calculate tumor volume using the formula: V = 1/2 (Length × Width^2^) [74]. Xenograft tumor experiments were performed according to the animal protocol approved by IACUC, which is in accordance with the guidelines established by the USPHS.

### Chromatin immunoprecipitation (ChIP) assays

ChIP assays were carried out as described previously [75]. The antibody (Thermo Fisher Scientific, Waltham, MA, USA) against human BRCA1 protein was used to immunoprecipitate chromatin DNA for quantitative ChIP assays. Mouse IgG was used a negative control. Immunoprecipitated chromatin DNA was assessed by qPCR using primers to amplify five different DNA regions (R1 to R5, shown in Figure 1G) upstream of the *NEAT1* gene. The primer sequences for amplifying these five different DNA regions upstream of the *NEAT1* gene can be found in the supplementary information. Results were normalized to input.

### Stem-cell sphere formation assays

Sphere formation assays were performed as previously described [76]. In brief, Cells were detached from culture plates using accutase (Biolegend, San Diego, CA, USA) and passed through a 25 G needle three times and a 40-μm strainer (Thermo Fisher Scientific) to obtain a single cell suspension. A total of 10,000 cells were seeded per well of a six-well plate coated with 2% polyhema (Sigma). After 7 days, spheres with size of ≥ 100 μm were counted.

### FACS analysis of surface antigen proteins

Fluorescence-activated cell sorting (FACS) analysis was performed using a FACSAria II cell sorter (BD Biosciences, San Jose, CA, USA). Cells were stained with the following antibodies from Biolegend: V450-conjugated anti-CD44, FITC-conjugated anti-CD44, PE-conjugated anti-CD49f, APC-conjugated anti-EpCAM and PE/Cy7-conjugated anti-CD24.

### Statistical analysis

Statistical analysis was performed by Student's *t* test. The *p* values of < 0.05 were considered significant. Data are presented as mean ± S.D. Data were analyzed using the GraphPad Prism software (version 6.0). The same software was also used in Regression analysis of gene expression correlation. The *p* values of < 0.05 from regression analysis were considered significant.

Other materials and general methods not included here can be found in the supplementary information.

## SUPPLEMENTARY MATERIALS FIGURES AND TABLE




