# Supplementary material for: Dysregulation of the BRCA1/long non-coding RNA NEAT1 signaling axis contributes to breast tumorigenesis
Source: Oncotarget. 2016 Aug 18;7(40):65067–89. doi: 10.18632/oncotarget.11364 (PMC5323139; doi:10.18632/oncotarget.11364)
Supplement: Supplementary file 1 [file oncotarget-07-65067-s001.pdf]

Dysregulation of the BRCA1/long non-coding RNA NEAT1 signaling axis contributes to breast tumorigenesis

Supplementary Materials

MATERIALS AND METHODS

Plasmid DNA construction

For constructing human NEAT1 expression plasmid, human Neat1 gene fragments were recovered from pCRII-topo-hNeat1 (Addgene, Cambridge, MA, USA) [1] and subcloned into the pZW1-sno vector to generate pZw1-sno-hNEAT1 [2].

Mouse Neat1 shRNAs were designed using the Biosettia's shRNA designer (http://biosettia.com/support/shrna-designer). The shRNA sequences were cloned into pLV-hU6-Ef1a-puro vector (Biosettia) according to the manufacturer's instructions. The scramble and two Neat1 shRNA sequences used in the study are: scramble, 5′-TAACTCGCTCGAAGGAATC-3′; shNeat1-1, 5′-GCC ACATTAATCACAACTT-3'; shNeat1-2, 5′- GCCACTGA ATACATCCATT-3'.

Plasmid DNA transfection

Plasmid DNA transfection was performed using Lipofectamine 2000 (Invitrogen) or FuGENE HD (Promega, Madison, WI, USA) according to the manufacturer's instructions. To obtain stable shRNA transfectants, transfected cells were selected in the culture medium containing 0.5 μg/ml of puromycin.

Quantitative RT-PCR analysis

qRT-PCR analysis of mRNA/miRNA/lncRNA expression was performed as described previously with normalization to either GAPDH or β-actin for mRNAs/lncRNAs and to U6 small nuclear RNA for miRNAs [3]. Human *NEAT1* gene is transcribed into two RNA isoforms, NEAT1-1 (~3.7 kb) and NEAT1-2 (~23 kb). Given that only NEAT1-1 contains a poly(A) tail, we used both oligo-dT and random hexamer primers to prepare two kinds of reverse transcription (RT) mixtures for allowing us to detect each NEAT1 RNA isoform level and also the total level of both isoforms. To measure the total level of both NEAT1 RNA isoforms, qRT-PCR was performed on RT mixtures generated from random hexamer primers using NEAT1-specific primers that can detect both NEAT1 isoforms (forward primer: 5′-TTTGTGCTTGGAACCTTGCT-3′; reverse primer: 5′-TCAACGCCCCAAGTTATTTC-3′). To quantify the expression level of NEAT1-1, qRT-PCR was performed on RT mixtures generated from oligo-dT primers using NEAT1-specific primers shown above. For the measurement of the NEAT1-2 expression level, qRT-PCR was performed on RT mixtures generated from random hexamer primers using NEAT1-2-specific primers (forward primer: 5′- TCTCCATTTCCCCATCTGAG-3′; reverse primer: 5′- CAGCCACAGAAAAGGGAGAG-3′). For miR-129-5p expression measurement, we used miScript primers and miScript-related reagents (Qiagen, Chatsworth, CA, USA) for RT and qRT-PCR assays.

Western blot analysis

Protein expression was examined by Western blotting using rabbit polyclonal antibodies for detecting BRCA1 (C-20), WNT4 (C-14) and β-catenin (H-102) (Santa Cruz Biotechnology, Santa Cruz, CA, USA). Mouse monoclonal antibody (Sigma, St Louis, MO, USA) was used to detect β-actin. Protein expression was detected by chemiluminescence (ECL, Amersham Biosciences; Arlington Heights, IL, USA). Expression of β-actin was used as a protein loading control.

DIG labeling of the ISH probe

To prepare the digoxigenin (DIG)-labeled probe for *in situ* hybridization analysis of mouse Neat1 RNA expression in mouse tissue, we performed PCR on mouse tail genomic DNA using a Neat1-specific primer pair (forward: 5′-CGGCTGTGAATGTTCCAGATG-3′; reverse: 5′-TCA ACCACCCAGTATCA AATCCA-3′) to amplify a 574- bp DNA probe. The PCR-amplified Neat1 DNA probe was subcloned into the pGEM-T-Easy vector (Promega). *In vitro* transcription was performed on pGEM-T-Neat1 using T7 or SP6 RNA polymerase based on the orientation of the inserted DNA to synthesize the DIG-labeled antisense Neat1 RNA probe. DIG RNA Labeling Kit (SP6/T7) (Roche, Indianapolis, IN, USA) and manufacturer's methods were used for the *in vitro* transcription synthesis of the DIG-labeled RNA probe.

In situ hybridization analysis

In situ hybridization was performed using the DIG-labeled Neat1 RNA probe. Briefly, tissue sections were

deparaffinized, then fixed with 4% paraformaldehyde and treated with proteinase K. After 4 hours of pre-incubation in a hybridization buffer at room temperature, tissue sections were hybridized with the Neat1 probe (25 ng/ml) in a hybridization buffer overnight at 55°C. After incubation in the 0.2 × SSC buffer for 1 hour at 60°C and treatment with peroxidase, tissue sections were blocked (10% FBS in 1 × PBS) for 1 hour at room temperature and then incubated with the anti-DIG antibody (1:500) overnight at 4°C. After PBST (1 × PBS with 0.05% Tween 20) wash, for the colorimetric detection reaction, tissue sections were incubated in the developer solution (NBT/BCIP, Roche) overnight at room temperature in dark. Slides were then counterstained with hematoxylin and mounted using D.P.X Mounting medium (Sigma). Stained tissue sections were visualized with Nikon Eclipse Ti microscope (Nikon Instruments Inc.; Melville, NY, USA).

ChIP primer sequences

The primer set 1 for R1:
forward: 5′-GCCCAGAAACAGCACTACAG-3′;
reverse: 5′-CTTATGAAACCAATGGAGGAGTC-3′
The primer set 2 for R2:
forward: 5′-CCTGCCTGCTGATACCACCTC-3′;
reverse: 5′-TAACACCCACACCCCATACAAAAC-3′
The primer set 3 for R3:
forward: 5′-CTGTCCCTCGGCTATGTCAGA-3′;
reverse: 5′-GGGGTAGAACATTCAAGAAGTGTG-3′
The primer set 4 for R4:
forward: 5′-CACGTCCCCTCGCAGCAC-3′;
reverse: 5′-CGGCAGGACATCTGGAAAATTC-3′
The primer set 5 for R5:
forward: 5′-GTGGCCGTGGAGGAATCGT-3′;
reverse: 5′-ATCCCTCCCTGTCGCTAACTC-3′

MicroRNA PCR array assays

Total RNA was isolated using the RNeasy Mini kit (Qiagen, Chatsworth, CA, USA). MicroRNA expression profiling was carried out using the Breast Cancer miScript miRNA PCR Arrays according to the manufacturer's instructions (Qiagen, Chatsworth, CA, USA). Real-Time PCR was performed using the SYBR Green PCR Master Mix (Qiagen) in a BioRad CFX 1000 real time PCR machine (BioRad, Hercules, CA, USA). The exported Ct data were analyzed using Excel-based PCR Array Data Analysis Templates (Qiagen). The average Ct value of each gene obtained from duplicate experiments was used to calculate its expression value, which is expressed as $2^{-\Delta Ct}$ ($\Delta Ct = Ct^{MOI} - \text{Ave } Ct^{ISR}$; MOI: miRNA of interest, ISR: the internal small RNA reference, Ave Ct^{ISR}: the average Ct value of five internal miRNA RNA references).

Reporter plasmid DNA construction and dual reporter assays

To construct the human WNT4 3′-UTR reporter plasmid, a fragment (1.0 kb) of the 3′-UTR of the human *WNT4* gene was amplified by PCR using primers 5′- ACG TTT GCT AGC TGG GGC TCT AAG TTT CAG GT-3′ and 5′- ACG TTT CTC GAG AGT CAC TGT CAC AAT TGC AAG A-3′ and cloned into the NheI and XhoI sites of the pSGG vector. The resulting reporter plasmid was then used as a template to generate a mutant reporter plasmid construct with mutations at the site recognized by miR-129-5p using the site-directed mutagenesis system (ThermoFisher Scientific, Grand Island, NY, USA) and primers 5′- TGC TAG TGA GGC ATA GTA TTT TAT ATC GGC TTA GTA GAC AGC TGA ACA G-3′ and 5′-CTG TTC AGC TGT CTA CTA AGC CGA TAT AAAata CTA TGC CTC ACT AGC A-3′. The mutant that contains three point mutations CTACTAA GCC(C to G)A(A to T) A(A to T)AA was confirmed by sequencing.

Bisulfite sequencing analysis

Genomic DNA was isolated using the QIAamp DNA Blood Mini Kit (Qiagen) according to the manufacturer's user manual. Isolated genomic DNA was bisulfite-converted using the EpiTect Bisulfite kit (Qiagen) according to the protocol described in the manufacturer's user manual. For bisulfite sequencing analysis of the *miR-129* gene, a 392-bp DNA fragment in the CpG-island region of *miR-129* was amplified from bisulfite-converted DNA using primers: 5′- GAA TTT TGA TAG GGA GAT AGA GGG A-3′ (forward) and 5′- ACT ATT AAA TTA TAT ACA ACA AAC CCA AAC-3′ (reverse). The PCR was carried out using the PCR cycle condition: 95°C, 30 sec; 57°C, 30 sec; 72°C, 60 sec (35 cycles). The PCR products were purified using the Qiagen PCR purification kit (Qiagen) and sequenced by using the sense primer with an Applied Biosystems automated fluorescent sequencer according to the manufacturer's instructions.

REFERENCES

1. Clemson CM, Hutchinson JN, Sara SA, Ensminger AW, Fox AH, Chess A, Lawrence JB. An architectural role for a nuclear noncoding RNA: NEAT1 RNA is essential for the structure of paraspeckles. Mol Cell. 2009; 33:717–726.
2. Yin QF, Hu SB, Xu YF, Yang L, Carmichael GG, Chen LL. SnoVectors for nuclear expression of RNA. Nucleic Acids Res. 2015; 43:e5.
3. Li Q, Eades G, Yao Y, Zhang Y, Zhou Q. Characterization of a stem-like subpopulation in basal-like ductal carcinoma *in situ* (DCIS) lesions. J Biol Chem. 2014; 289:1303–1312.

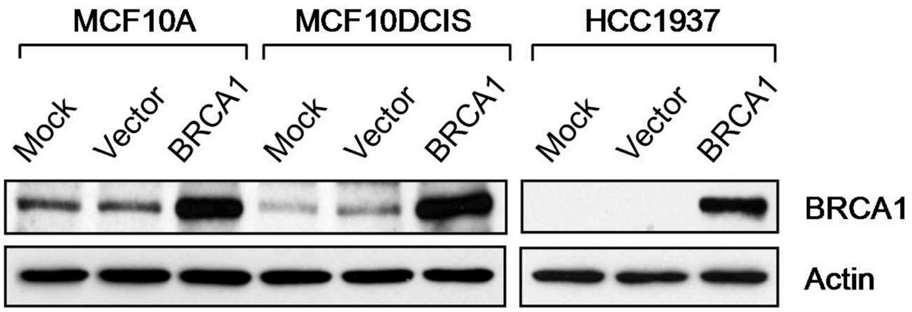

Supplementary Figure S1: Western blot analysis of BRCA1 protein levels in MCF10A, MCF10DCIS and HCC1937 cells transfected with BRCA1 expression plasmid DNA. MCF10A, MCF10DCIS and HCC1937 cells were transfected with either the empty vector control or BRCA1 expression plasmide DNA. 48 hours after transfection, transfected and mock-treated cells were harvested and then subjected to Western blot analysis of BRCA1 and β-actin protein levels. Actin levels serve as a protein loading control.

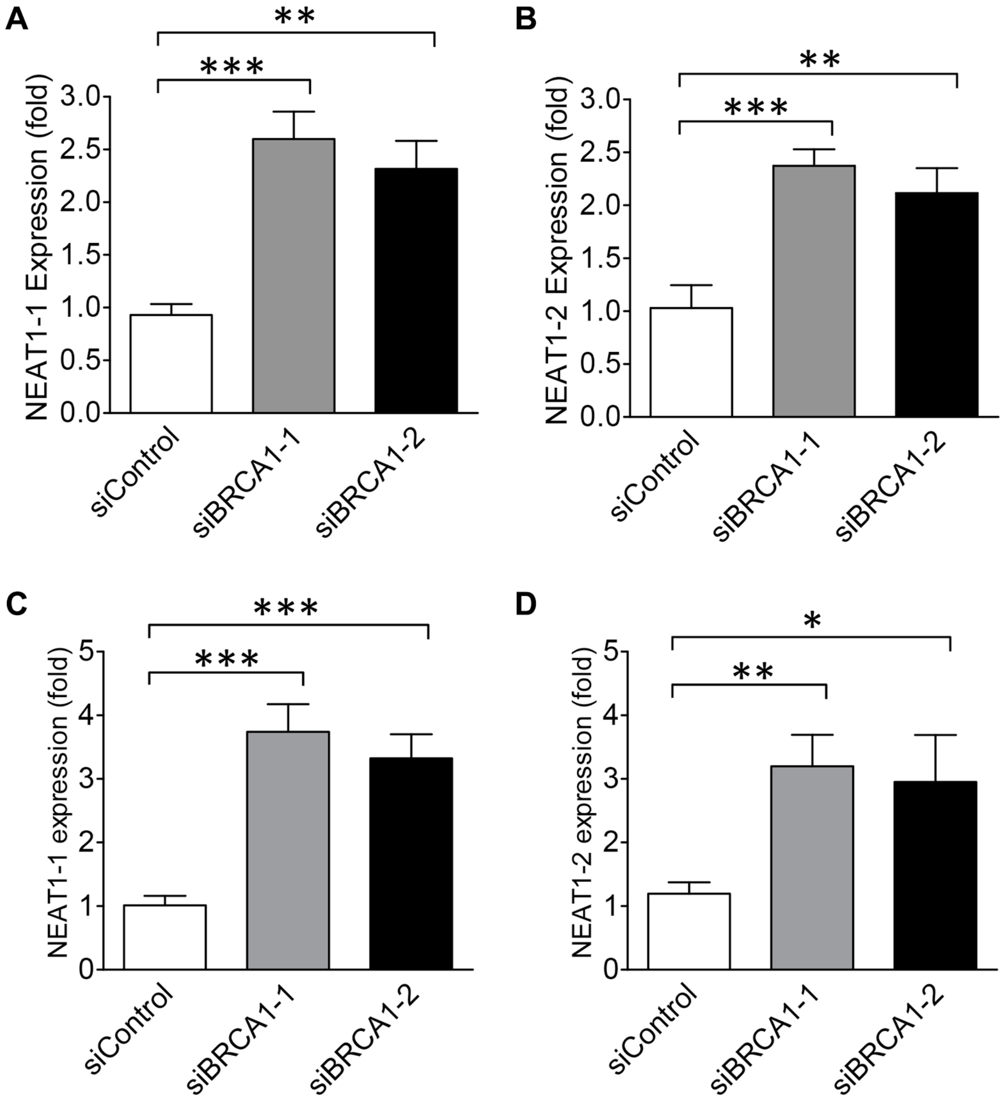

Supplementary Figure S2: Quantitative RT-PCR analysis of expression levels of two NEAT1 isoform RNAs in MCF10A and MCF7 cells transfected with two different BRCA1 siRNAs. MCF10A (**A**, **B**) and MCF7 (**C**, **D**) cells were transfected with either the scramble control or BRCA1 siRNAs. 48 hours after transfection, siRNA-transfected cells were harvested and then subjected to qRT-PCR analysis of NEAT1-1 (3.7 kb isoform RNA) (A, C) and NEAT1-2 (~23 kb isoform RNA) (B, D) expression levels. $*p < 0.05$; $**p < 0.01$; $***p < 0.001$.

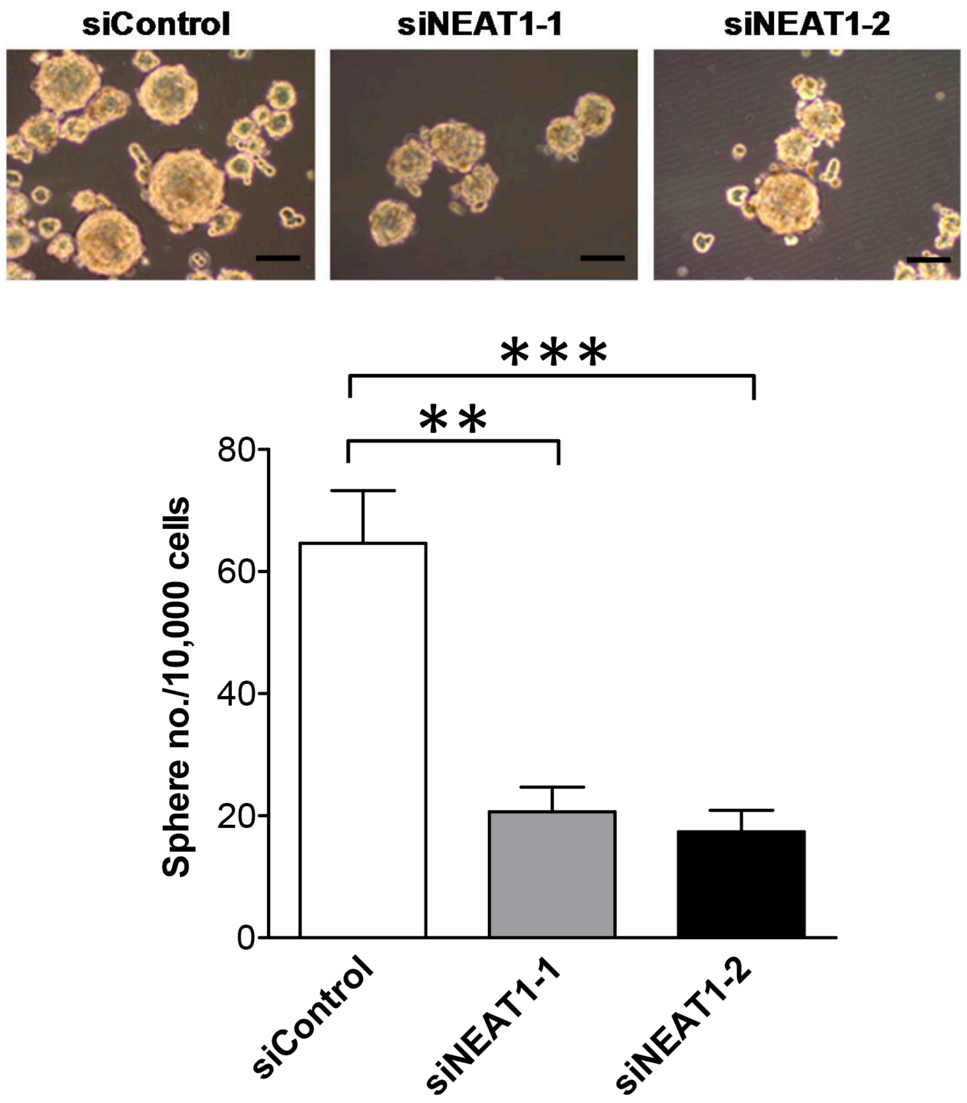

Supplementary Figure S3: Knockdown of NEAT1 by siRNA leads to suppression of sphere formation of HCC1937 cells. HCC1937 cells were transfected with two effective NEAT1 siRNAs and the scramble control (siControl) individually. At 24 hours after siRNA transfection, transfected cells were harvested and then subjected to sphere formation assays. The pictures of formed spheres are shown in the top panel and the counted sphere number data were plotted to make a bar graph that is shown in the bottom panel. The scale bar shown in sphere pictures indicates 100 μm. The error bar represents the SD of the dataset ($n = 3$). $^{**}p < 0.01$, $^{***}p < 0.001$.

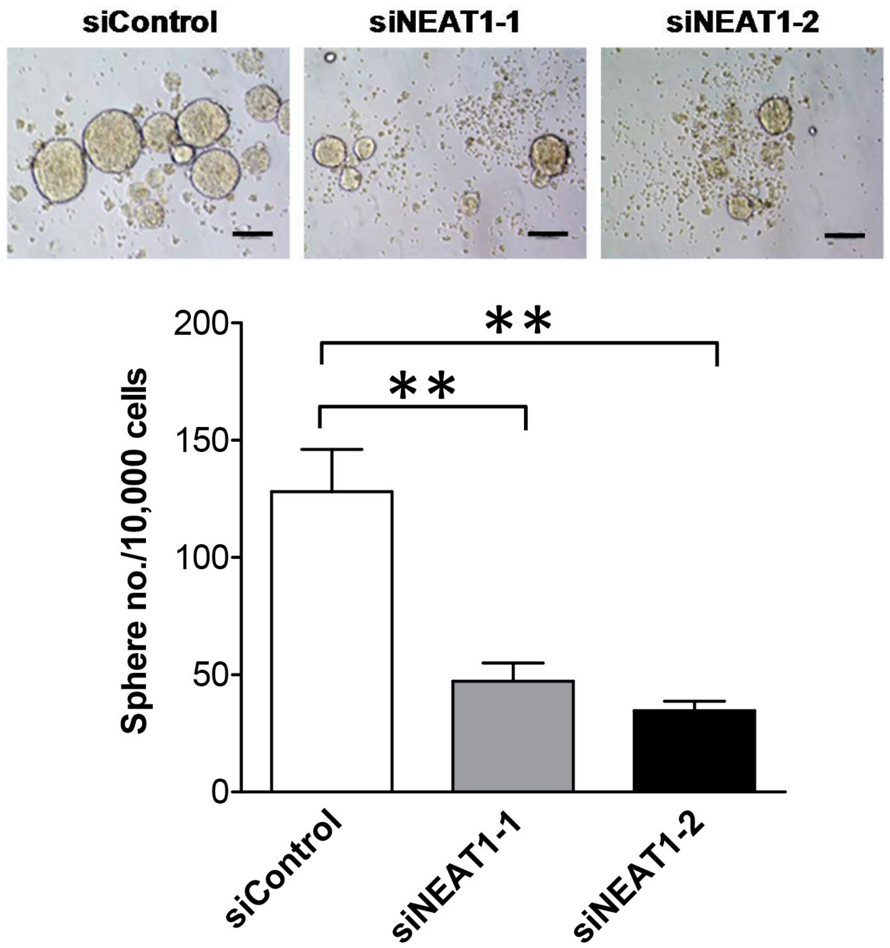

Supplementary Figure S4: Knockdown of NEAT1 by siRNA leads to suppression of sphere formation of MCF7 cells. MCF7 cells were transfected with two effective NEAT1 siRNAs and the scramble control (siControl) individually. At 24 hours after siRNA transfection, transfected cells were harvested and then subjected to sphere formation assays. The pictures of formed spheres are shown in the top panel and the counted sphere number data were plotted to make a bar graph that is shown in the bottom panel. The scale bar shown in sphere pictures indicates 100 μm. The error bar represents the SD of the dataset ($n = 3$). $^{**}p < 0.01$.

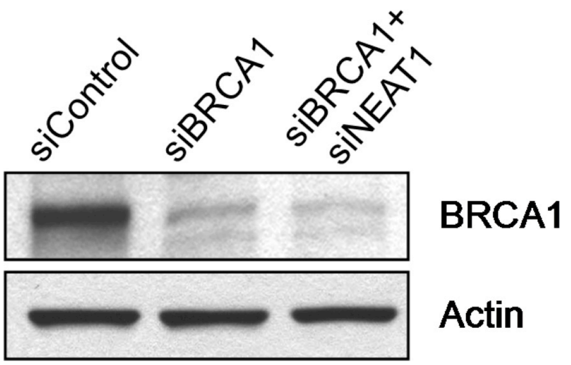

Supplementary Figure S5: The knockdown efficacy of the BRCA1 siRNA is not affected by co-transfection with the NEAT1 siRNA. Western blot analysis of BRCA1 and β-Actin was performed on MCF10DCIS cells transfected with siControl, siBRCA1 or siBRCA1 plus siNEAT1.

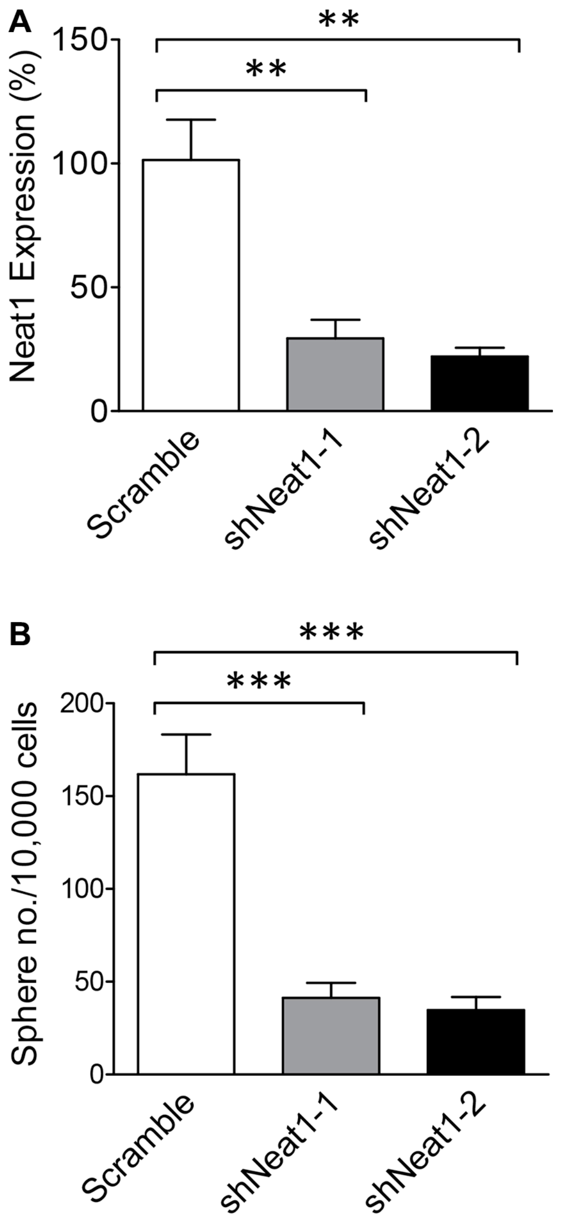

Supplementary Figure S6: Knockdown of Neat1 suppresses the sphere formation of CSCs in mouse *Brca1* mutant mammary tumors. (**A**) qRT-PCR analysis of the knockdown efficiency of Neat1 shRNAs. Primary tumor cell cultures isolated from Brca1-deficient mammary tumors developed in *MMTV-cre;Brca1*$^{co/co}$ mice were transfected with the scramble shRNA plasmid or two different Neat1 shRNA plasmids (shNeat1-1 and shNeat1-2) and transfected cell cultures were selected in the culture medium containing 0.5 µg/ml of puromycin. The knockdown efficiency of these two mouse Neat1 shRNAs was examined by qRT-PCR analysis of Neat1 expression normalized by actin expression. (**B**) Neat1 knockdown inhibits the self-renewal of CSCs in Brca1-deficient mammary tumors. Stable scramble shRNA-expressing and shNeat1-expressing Brca1-deficient mammary tumor cell cultures were subjected to the sphere formation assay. $^{**}p < 0.01$; $^{***}p < 0.001$.

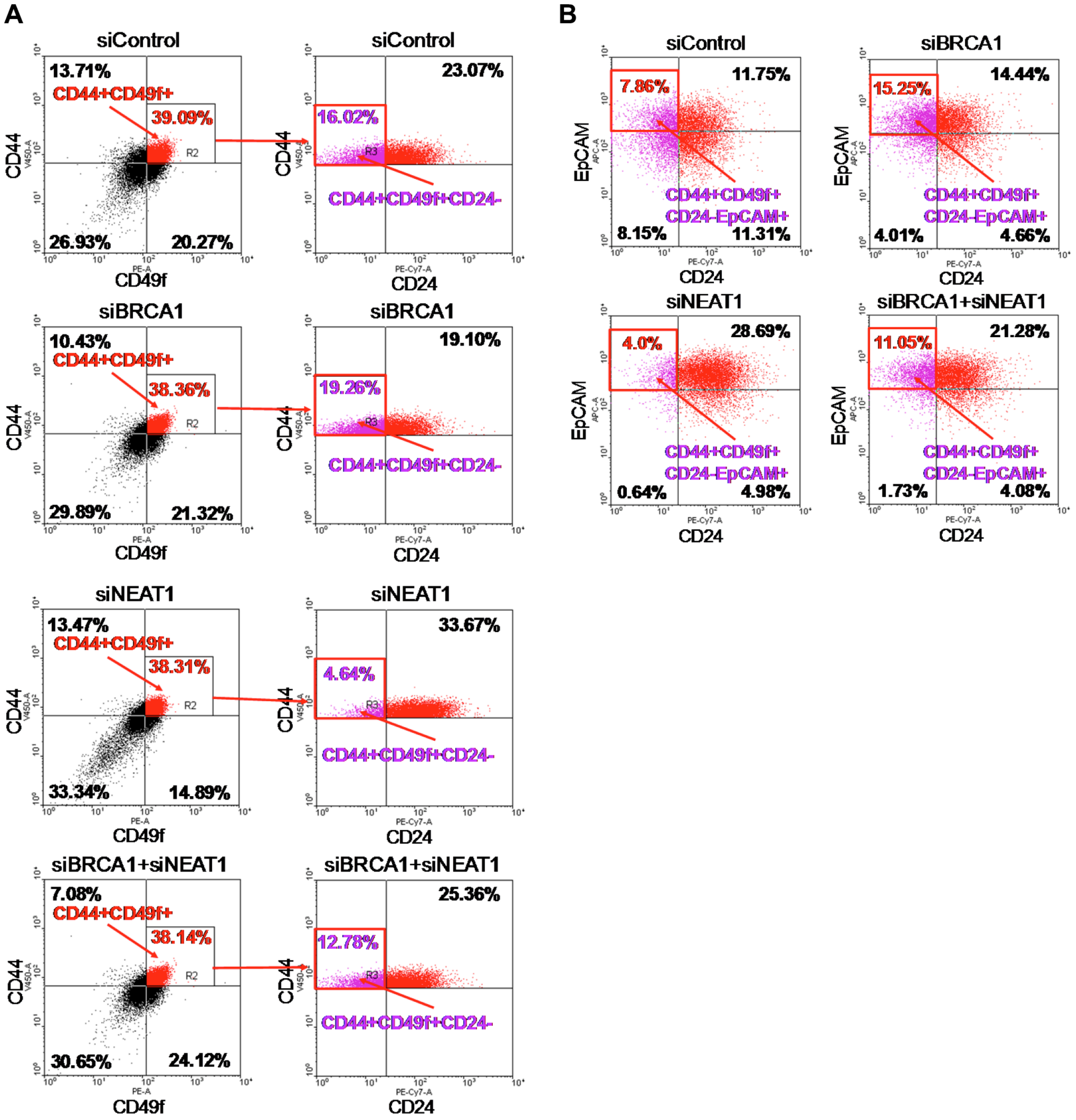

Supplementary Figure S7: Co-knockdown of NEAT1 attenuates the expanded EpCAM+BCSC population in BRCA1-knockdown MCF10DCIS cells. (**A**) Flow cytometry analysis of surface antigen markers CD44, CD49f and CD24 for BCSC identification was performed on siRNA-transfected MCF10DCIS cells as indicated. The 2D dot plots that profile CD44 and CD49f are shown in the left panel. The gated CD44+CD49f+ cell subsets were subjected to the 2D dot plot analyses that profile CD44 and CD24, which are shown in the right panel. The BCSC-enriched cell subset CD44+CD49f+CD24– is depicted by the purple color. (**B**) Flow cytometry analysis of BCSC-related protein antigens CD44, CD49f, CD24 and EpCAM was performed on siRNA-transfected MCF10DCIS cells shown in (A). The gated CD44+CD49f+ cell subsets shown in the left panel of (A) were subjected to the 2D dot plot analyses to profile CD24 and EpCAM. The BCSC-enriched cell subset CD44+CD49f+CD24– is depicted by the purple color. The expanded EpCAM+BCSC-enriched cell subset (CD44+CD49f+CD24–EpCAM+) by BRCA1 knockdown was gated and its percentage is shown in the dot plot.

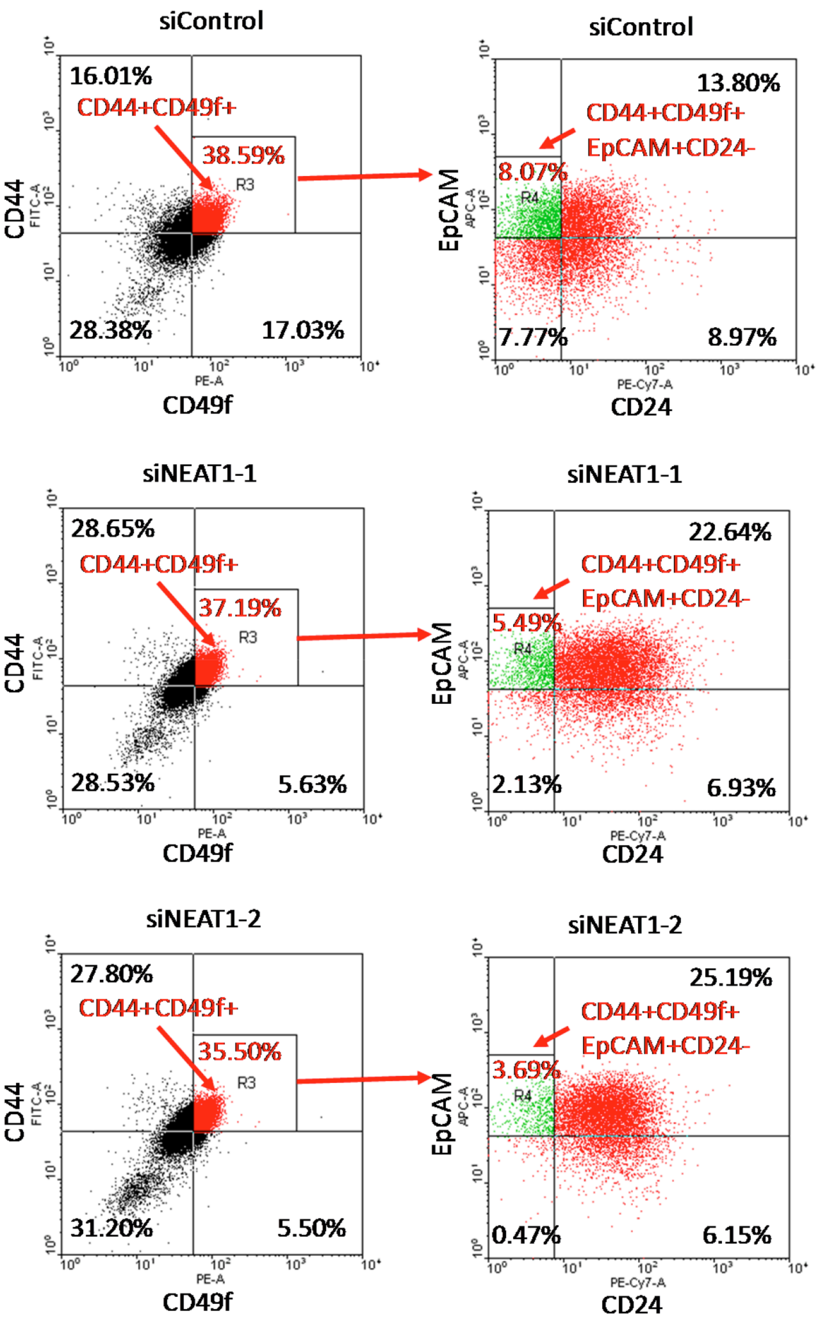

Supplementary Figure S8: NEAT1 knockdown results in a decrease in the EpCAM+ BCSC population (CD44+CD49f+CD24-EpCAM+) in MCF10DCIS cells. Flow cytometry analysis of stem-cell-related protein antigens CD44, CD49f, CD24 and EpCAM was performed on MCF10DCIS cells transfected with NEAT1 siRNAs or the scramble RNA control (siControl). The 2D dot plots that profile CD44 and CD49f are shown in the left panel. The gated CD44+CD49f+ cell subsets were subjected to the 2D dot plot analyses that profile CD24 and EpCAM, which are shown in the right panel. The EpCAM+BCSC-enriched cell subset CD44+CD49f+CD24–EpCAM+ is depicted by the green color. The percentages of gated cells are indicated in FACS dot plots.

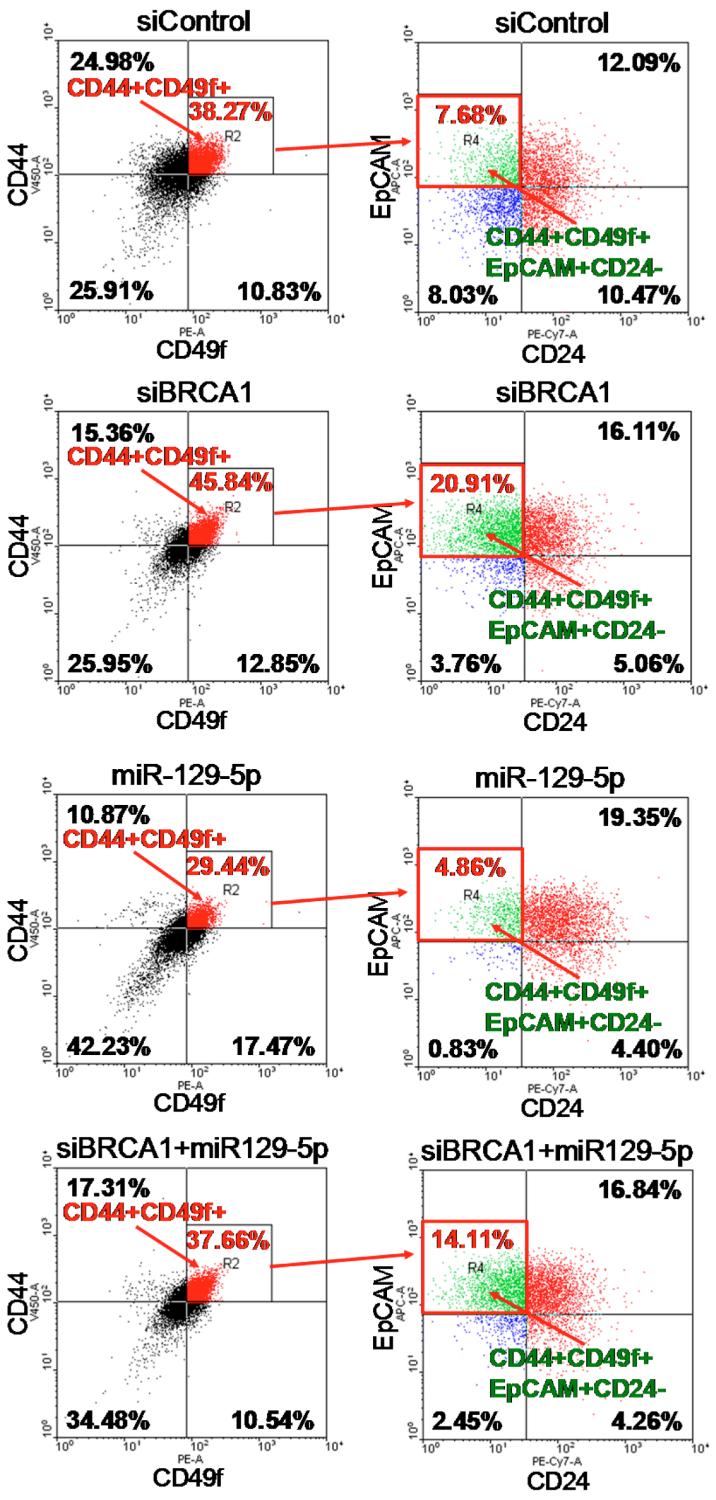

Supplementary Figure S9: Flow cytometry analysis of the impact of the miR-129-5p mimic on the BCSC population in MCF10DCIS cells with or without BRCA1 knockdown. Flow cytometry analysis of BCSC protein antigens CD44, CD49f, CD24 and EpCAM was performed on transfected MCF10DCIS cells 72 hours post-transfection as indicated. The gated CD44+CD49f+ cell subsets shown in the left panel were subjected to the 2D dot plot analyses that profile CD24 and EpCAM. As mentioned in Supplementary Figure S7B, the CD44+CD49f+CD24–EpCAM+ (EpCAM+BCSC) cell subset (the gated green cells) was expanded by BRCA1 knockdown.

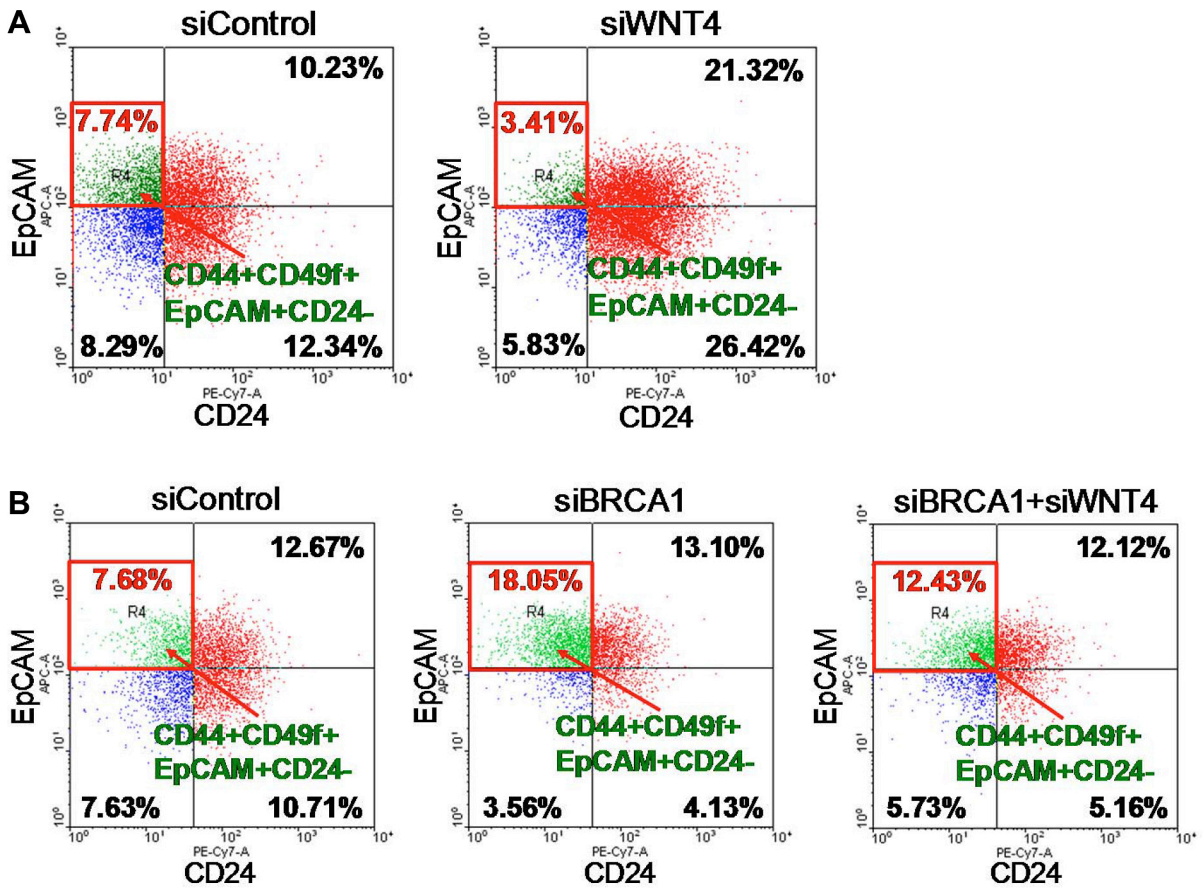

Supplementary Figure S10: WNT4 is functionally involved in promoting the stemness of BRCA1-deficient MCF10DCIS cells. (**A**) WNT4 inactivation reduces the EpCAM+BCSC population in MCF10DCIS cells. Flow cytometry analysis of four stem-cell-related protein antigens CD44, CD49f, CD24 and EpCAM was performed on MCF10DCIS cells transfected with either the control or WNT4 siRNA. The gated CD44+CD49f+ cell subsets were subjected to the 2D dot plot analyses that profile CD24 and EpCAM. The percentage of the CD44+CD49f+CD24– cell subset is the sum of the percentages of CD44+CD49f+CD24-EpCAM+ (the green cell subset) and CD44+CD49f+CD24–EpCAM– (the blue cell subset). (**B**) Co-knockdown of WNT4 attenuates the BRCA1-knockdown-induced expansion of EpCAM+BCSCs in BL-DCIS tumor cells. MCF10DCIS cells were transfected with the control, BRCA1 siRNA, or combined siRNAs targeting both *BRCA1* and *WNT4* genes. At 48 hrs after transfection, siRNA-transfected MCF10DCIS cells were subjected to the same flow cytometry analysis as described in (A).

Supplementary Table S1: The expression information for BRCA1, NEAT1 and miR-129-5p in 38 breast cancer cell lines. See Supplementary_Table_S1
